# Bile signalling promotes chronic respiratory infections and antibiotic tolerance

**DOI:** 10.1038/srep29768

**Published:** 2016-07-19

**Authors:** F. Jerry Reen, Stephanie Flynn, David F. Woods, Niall Dunphy, Muireann Ní Chróinín, David Mullane, Stephen Stick, Claire Adams, Fergal O’Gara

**Affiliations:** 1 BIOMERIT Research Centre, School of Microbiology, University College Cork, National University of Ireland, Cork, Ireland; 2 Paediatric Cystic Fibrosis Unit, Cork University Hospital, Cork, Ireland; 3 Telethon Kids Institute, Perth, Western Australia; 4 School of Biomedical Sciences, Curtin Health Innovation Research Institute, Curtin University, Perth, WA 6102, Australia

## Abstract

Despite aggressive antimicrobial therapy, many respiratory pathogens persist in the lung, underpinning the chronic inflammation and eventual lung decline that are characteristic of respiratory disease. Recently, bile acid aspiration has emerged as a major comorbidity associated with a range of lung diseases, shaping the lung microbiome and promoting colonisation by *Pseudomonas aeruginosa* in Cystic Fibrosis (CF) patients. In order to uncover the molecular mechanism through which bile modulates the respiratory microbiome, a combination of global transcriptomic and phenotypic analyses of the *P. aeruginosa* response to bile was undertaken. Bile responsive pathways responsible for virulence, adaptive metabolism, and redox control were identified, with macrolide and polymyxin antibiotic tolerance increased significantly in the presence of bile. Bile acids, and chenodeoxycholic acid (CDCA) in particular, elicited chronic biofilm behaviour in *P. aeruginosa*, while induction of the pro-inflammatory cytokine Interleukin-6 (IL-6) in lung epithelial cells by CDCA was Farnesoid X Receptor (FXR) dependent. Microbiome analysis of paediatric CF sputum samples demonstrated increased colonisation by *P. aeruginosa* and other Proteobacterial pathogens in bile aspirating compared to non-aspirating patients. Together, these data suggest that bile acid signalling is a leading trigger for the development of chronic phenotypes underlying the pathophysiology of chronic respiratory disease.

The societal challenge posed by the threat of a post antibiotic era is underpinned by the growing ineffectiveness of conventional antimicrobial therapies^1^. This challenge is further confounded by our lack of understanding of how microbial populations contribute to the pathophysiology of disease and in particular how chronic pathogenic species establish and persist within these communities. The conventional assessment of microbial pathogens at the site of infection has advanced in recent years to a growing acceptance of the importance of microbial communities, often referred to as microbiomes^2^. This is particularly true of chronic respiratory disease, which is fast becoming one of the most significant health and societal challenges facing an ageing global population.

A common thread among respiratory microbiome studies has been the emergence of signature microbial profiles linked to disease status. Characteristic microbiomes have been reported for Cystic Fibrosis (CF)^3^ and Chronic Obstructive Pulmonary Disease (COPD)^4^, while profiles for asthma and non-CF bronchiectasis are also emerging^5^,^6^. As expected, pathogens linked to the aetiology of the respective diseases have dominated much of the microbiome data. However, the presence of diverse communities of microorganisms co-habiting the clinical niche, has added a new dimension to the existing models of microbial infections. What has yet to be established is the key driver of these signature microbiomes; effectively the environmental trigger(s) governing the dynamics of these populations, and the emergence of dominant pathogenic species, many of which are considered opportunistic.

The aspiration of bile acids into the lungs of respiratory patients has emerged as a major co-morbidity of a range of respiratory diseases^7^,^8^,^9^,^10^, and in the case of CF has been shown to correlate with reduced biodiversity and the emergence of dominant Proteobacterial pathogens^10^,^11^. A correlation has also emerged between the presence of bile acids in the respiratory tract and increased inflammation^12^, possibly linked to the hypoxia inducible factor (HIF)-1^13^,^14^, a key master regulator involved in both the hypoxic and immune response of mammalian systems. The primary driver of bile aspiration in respiratory patients is a condition called gastro-oesophageal reflux disease (GERD). Owing largely to factors such as delayed gastric emptying, damage to the lower oesophageal sphincter, and in some cases chest physiotherapy, the leakage of bile into the oesophageal tract can occur in up to 80% of CF patients and 35% of COPD patients^15^,^16^. Indeed, these may be under-estimations of the prevalence of reflux and subsequent aspiration in light of the fact that asymptomatic or silent aspiration goes undetected in the clinic.

The emerging evidence for the impact of bile acids on the pathophysiology of respiratory disease strongly suggests that this host factor has the potential to offer a unifying principle for the onset of chronic infection and chronic inflammation in respiratory disease patients. In order to understand the mechanism involved in the chronic response of respiratory pathogens and host cells to aspirated bile, we undertook a systems based analysis, focusing initially on *P. aeruginosa*, the primary pathogen associated with morbidity and mortality in CF patients. An integrated approach using transcriptomics, functional genomics, and immunochemistry led to the identification of a network of genes linked to virulence, adaptive metabolism, and antibiotic tolerance, underpinning the bile response in both the pathogen and in host cells. Individual bile acids were sufficient to elicit the chronic response, with chenodeoxycholic acid (CDCA) emerging as a cross-kingdom signal interfacing with both pathogen and host. Longitudinal analysis of the paediatric CF microbiome revealed characteristic aspirating and non-aspirating profiles, while phenotypic analysis of the isolates from these patients revealed a largely conserved response to physiologically relevant concentrations of bile, although evidence of phenotypic heterogeneity was observed. These data suggest that bile acid signalling may play a previously unforeseen central role in disease progression and antibiotic tolerance in respiratory disease patients.

## Results

### *P. aeruginosa* develops a virulence and redox transcriptome signature in response to bile

**Figure 1:**
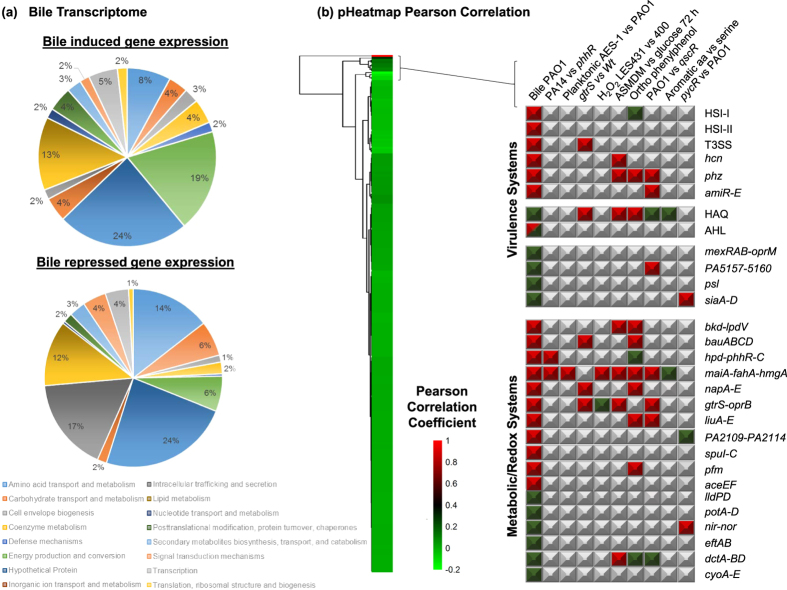
Bile transcriptional footprint. (**a**) Pie-chart profiling of the transcriptional response to bile in *P. aeruginosa*. (**b**) Singular global analysis of the bile transcriptome from the Pearson Correlation matrix using pheatmap. Correlation values are presented as per key. Following this, the clustered transcriptome profiles were interrogated for virulence and redox associated genes, representing a subset of the global profile. Red indicates downregulation of gene(s) within the designated system, while green denotes upregulation. The red/green coloured AHL box signifies *las* induction and *rhl* suppression. Grey boxes represent gene(s) or systems that are not significantly altered in the respective transcriptome datasets.

Transcriptome profiling of *P. aeruginosa* in the presence and absence of bile was undertaken to investigate the molecular mechanism underpinning the observed phenotypic bile response. In total 120 genes exhibited increased expression (ranging from 2.0 to 41.0 fold) in response to bile while a further 247 genes were downregulated under these conditions (ranging from −2.0 to −59.4 fold) (Fig. 1a, Supplementary Table S1 and Supplementary Fig. S1). Gene ontology and KEGG analysis of altered gene expression broadly revealed elevated levels of genes involved in energy production and metabolism, redox control, and cell envelope biogenesis, while those involved in intracellular trafficking and secretion as well as amino acid metabolism were suppressed in response to bile (Supplementary Table S1). Furthermore, several genes involved in the glyoxylate shunt pathway (*mqoB* and *glcB*), and the early stages of the TCA cycle (*acnA* and *idh*) were upregulated, while genes involved in glucose metabolism/glycolysis (*lpdV*, *gapA*, *glk*, and *aceEF*), the later stages of the TCA cycle (*PA0853-54* and *aspA*) and proton motive force (*pfm*) were all downregulated in the presence of bile. These systems coordinate cellular respiration and redox control within the cell, and their reorganisation suggests a form of adaptive metabolism in response to bile.

Consistent with the phenotypic changes previously observed in the presence of bile^11^, expression of genes associated with a switch from acute towards chronic behaviour and biofilm formation in *P. aeruginosa* was increased in response to bile. These include *pqsA* and *lasI* and *psl*, all of which have been previously shown to contribute to biofilm formation and virulence in *P. aeruginosa*. Previously, through promoter fusion and thin layer chromatographic analysis, we have shown increased production of the PQS and 3-oxo-C12 homoserine lactone quorum sensing signals in response to bile^11^. A downstream effect of this would appear to be increased elastolytic activity (Supplementary Fig. S1), which is known to be regulated by the PQS system^17^, and is secreted by over 75% of clinical *P. aeruginosa* isolates^18^. In addition, the increased *psl* expression was reflected in increased polysaccharide production in response to bile, as evidenced through congo red binding analysis (Supplementary Fig. S1). Transcriptional regulators that have been shown to play central roles in chronic associated phenotypes including biofilm formation (*psrA*), iron-scavenging and exopolysaccharide production (*ppyR*), oxidative stress response (*ohrR*), secretion and antibiotic tolerance (*mexR*) were all increased^19^,^20^,^21^,^22^ (Supplementary Table S1). Conversely, genes associated with acute infection, e.g. T3SS, hydrogen cyanide (*hcnABC*), amidase (*amiR)* and phenazine (*phzA-E*) were repressed in bile treated cells, while transcriptional regulators linked to acute virulence phenotypes such as choline (*gdbR*) and isoprenoid (*gnyR*) degradation and T6SS HSI-II expression (*sfa2*)^23^,^24^,^25^ also exhibited decreased expression in response to bile.

### Meta transcriptional analysis reveals a bile-signature response

In order to more comprehensively elucidate the transcriptional mechanism through which bile elicits a chronic response, a database of 250 *P. aeruginosa* transcriptome datasets was created using publically available transcriptome profiles. The database was converted to a Pearson correlation matrix heatmap, which provided a unique framework for the interrogation of transcriptomic profiles for this organism (Fig. 1b and Supplementary Table S2). The bile signature in *P. aeruginosa* was most closely linked to a number of virulence and metabolism/redox associated transcriptome profile datasets, particularly (a) a *phhR* mutant vs PAO1 (b) a *gtrS* mutant vs PAO1 and (c) Australian Epidemic Strain (AES-1) vs PAO1. The PhhR phenylalanine catabolic regulon^26^ was suppressed in the presence of bile, including *fahA* and *hmgA*, *hpd*, and *phhABC* (Supplementary Table S1), while the GtrS-GltR (Fig. 1b) two component system involved in glucose/ketogluconate metabolism has recently been shown to control T3SS in response to external triggers through modulation of central metabolic pathways and small RNA expression^27^. The bile transcriptome profile was inversely related to (a) aromatic amino acids vs serine, (b) the *pycR* mutant vs PAO1, and (c) ASMDM vs glucose OD_600 nm_ 0.3. The altered expression of genes involved in cellular metabolism, transport, and phenazine production was common to many of the transcriptional profiles that cluster with the bile transcriptome.

A more focused analysis of virulence and metabolic systems in the 9 transcriptomes that clustered closely with the bile profile revealed a unique bile-specific response (Fig. 1b). In spite of considerable overlap in the total profile of altered gene expression between each of the selected transcriptomes, the majority of the changes in secretion systems, virulence and redox gene expression were almost entirely specific to the bile transcriptome. Genes associated with chronic infection, e.g. *psl* (biofilm formation), *mexAB-OprM* (antibiotic tolerance), and *siaA-D* (transition to a sessile lifestyle through cyclic-di-GMP synthesis) were all increased in the bile transcriptome, but unaltered or downregulated in the other clustered profiles (Fig. 1b). Similarly, genes associated with acute virulence were for the most part only altered in the bile transcriptome, with the exception of the *phz* cluster. Therefore, the meta-analysis based approach afforded by the database provides further evidence that bile may be a global molecular signal that governs the switch from acute to chronic biofilm growth in *P. aeruginosa* in the lung, potentially through changes in cellular respiration and redox control.

### Bile exerts a negative influence on cellular respiration and redox potential

The reorganisation of cellular metabolic pathways indicated by the transcriptome profiling was considered likely to result in changes in redox potential within the cell, particularly the apparent metabolic flux away from glucose catabolism. To test this hypothesis, a tetrazolium violet redox assay was performed^28^. This assay, which is used widely in microbial physiology studies, indirectly measures the change that occurs in the key NAD^+^/NADH redox-modulating couple in the presence and absence of bile. These redox carriers play an important role in cellular respiration and there is a growing appreciation that adaptive metabolism is intrinsically linked to virulence and pathogenesis in a broad range of microbial organisms^27^,^29^,^30^,^31^,^32^,^33^.

**Figure 2:**
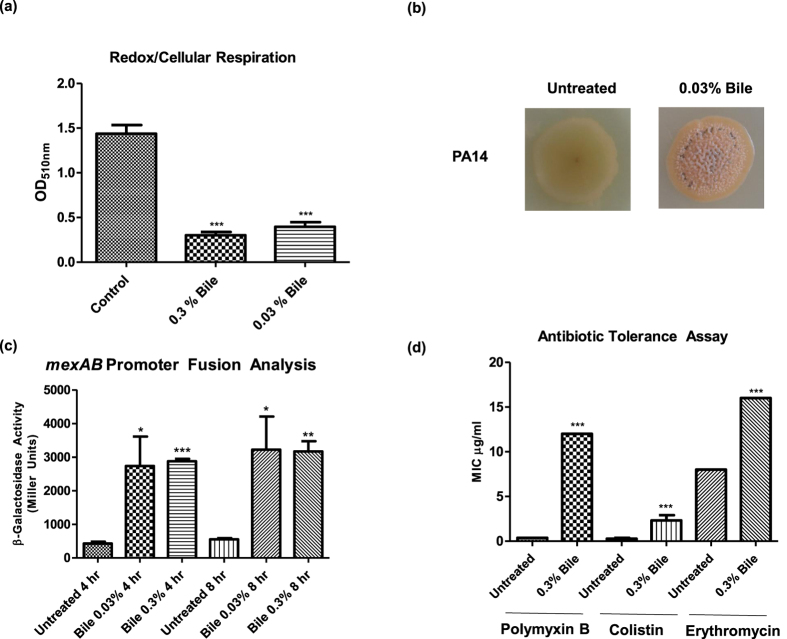
Bile suppresses redox and promotes antibiotic tolerance. (**a**) Physiologically relevant concentrations of bile elicit a strong suppression of redox activity in *P. aeruginosa*. (**b**) Morphological changes consistent with redox flux with wrinkled colony formation on bile treated TSA plates. (**c**) Increased *mexAB* promoter activity in the presence of bile. (**d**) E-strip antibiotic tolerance assays reveal bile-dependent tolerance to polymyxin and macrolide antibiotics in the presence of bile. Data is the mean of at least three independent biological replicates. Statistical analysis was performed by Student’s t-test (***p ≤ 0.001).

Significant suppression of cellular respiration in *P. aeruginosa* was observed in the presence of bile when compared to untreated samples (Fig. 2a). Previously, changes in redox status have been linked to altered colony morphology, where bursts of activity have been demonstrated to result in wrinkly biofilm-like formation patterns developing on the surface of agar plates^34^. This hallmark transition was observed in *P. aeruginosa* colonies on bile treated TSA plates but not on the untreated equivalent controls (Fig. 2b), further suggesting that changes in redox are an integral effect of bile on *P. aeruginosa*. Clues as to the mechanism through which the redox state is altered in the cell lie within the transcriptome footprint, and amongst other approaches metabolomics based analysis will ultimately be necessary to fully elucidate these pathways.

### Bile promotes antibiotic tolerance independent of MexAB-OprM

A key phenotype linked to chronic infections is the increased tolerance to antibiotic challenge. Once in the biofilm state, many microbial infections become refractory to conventional antibiotics. Consistent with the switch towards a chronic lifestyle, the expression of several multidrug resistance systems was found to be increased in response to bile (Supplementary Table S1). This included upregulation of genes encoding the MexAB-OprM system, confirmed by promoter fusion assays (Fig. 2c). The MexAB system is analogous to the AcrAB bile inducible efflux pump responsible for tetracycline, chloramphenicol, ampicillin, nalidixic acid, and rifampin resistance in *E. coli*^35^. Bile also increased expression of the *PA5157–5159* genes encoding components of the uncharacterised PA5158-PA5160 putative multidrug efflux pump, homologous to the ErmAB efflux system from *E. coli*^36^. Furthermore, upregulation of *PA3310* (*PA14_21210*) was notable for its recently uncovered role in polymyxin tolerance^37^. The upregulation of these and other efflux systems prompted us to test a panel of antibiotics for *P. aeruginosa* tolerance in the presence and absence of bile.

Using a combination of E-strips, antibiotic disks, and growth kinetic assays, bile was found to enhance tolerance of *P. aeruginosa* to the three key clinical antibiotics; colistin, polymyxin B, and erythromycin in the wild-type strain (Fig. 2d). Previously, *P. aeruginosa* has been shown to modify its Lipid A core in response to polymyxin challenge through either 4-amino-4-deoxy-l-arabinose (Ara4N) or phosphoethanolamine (pEtN) modification through PA14_21210 (PA3310) and other factors^38^. Although considered refractory to transmissible resistance, recent reports of plasmid-encoded polymyxin resistance systems linked to modification of the cell membrane have emerged^39^. However, the increased polymyxin B tolerance in the presence of bile was comparable between wild-type and an isogenic PA14_21210 TnM, while disk assays revealed that tolerance to erythromycin was independent of both *mexAB* and *PA5157–5160* (Supplementary Fig. S2).

### CDCA signals a chronic infection response in both pathogen and host

**Figure 3:**
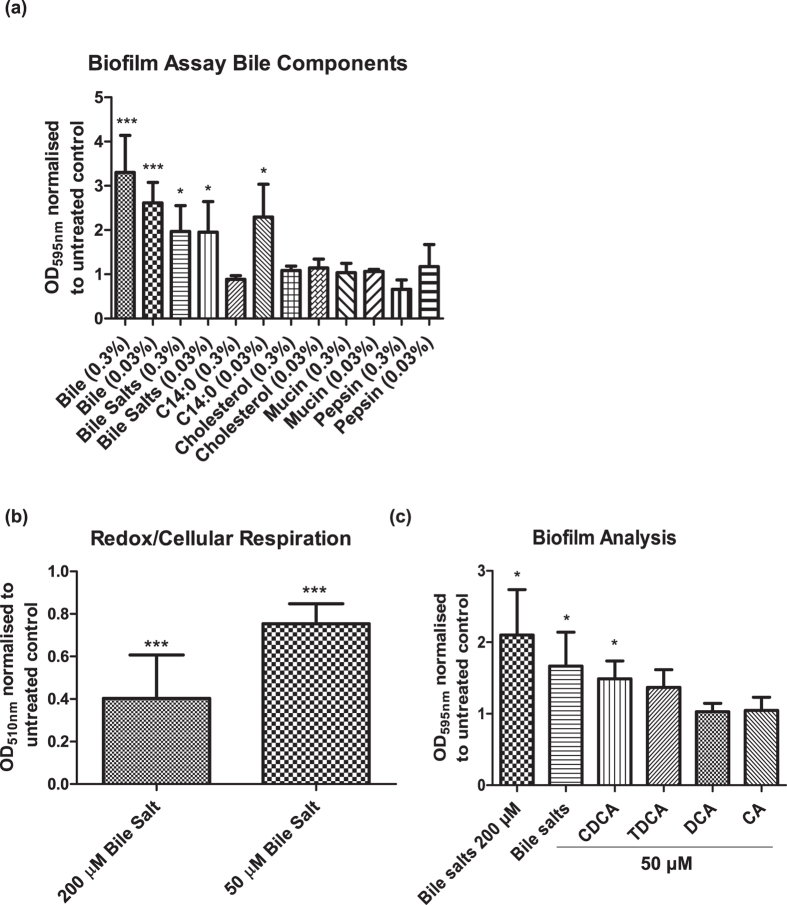
Bile salts elicit chronic lifestyle in *P. aeruginosa.* (**a**) Bile salts trigger biofilm formation in *P. aeruginosa.* The 0.03% solution of bile salts is equivalent to approximately 300 μM. (**b**) Bile salts at 50 μM also suppress the redox response. (**c**) CDCA at 50 μM was capable of enhancing biofilm formation in *P. aeruginosa*. In each panel, data is presented as OD(_595 nm or 510 nm_) normalised to the untreated control. All experiments are the average or representative of at least three independent biological replicates. Statistical analysis was performed by Student’s t-test, one-way ANOVA or Bootstratio (*p ≤ 0.05, **p ≤ 0.005, ***p ≤ 0.001).

Bile is a complex mixture of organic and inorganic components, which includes cholesterol, fatty acids, pepsin and bile acids or salts. Therefore, phenotypic assays were undertaken to investigate the contribution of each compound to the chronic infection switch. Bile salts, at physiological concentrations of 50 μM^9^,^10^,^12^, were shown to increase biofilm formation. In contrast, addition of other bile components such as cholesterol, pepsin, or long chain fatty acids did not significantly enhance this chronic associated phenotype (Fig. 3). An exception to this was the activity of C14:0 (myristic acid) which was the sole long chain fatty acid (LCFA) capable of increasing biofilm formation in *P. aeruginosa* (Fig. 3a and Supplementary Fig. S3). However, C14:0 did not significantly affect swarming motility comparable to bile salts, nor did it enhance PQS production (data not shown) and therefore was not considered further. Bile salts were also found to be responsible for the suppression of cellular respiration in *P. aeruginosa* (Fig. 3b), further underpinning their role in modulating pathogen behaviour in the lungs of aspirating CF patients.

The activity of bile acids within the host are known to be structure specific, whereby different bile acids can elicit very different responses^14^. Therefore, to investigate whether the same principle would apply in the microbial response to bile, 12 prevalent human bile acids were tested for their biofilm activity in *P. aeruginosa*. Interestingly, a subset were found to elicit biofilm formation similar to the complex bile salt mixture, including GCA, LCA, TLCA and CDCA, with only sodium chenodeoxycholate (CDCA) producing a statistically significant response (Fig. 3c). This supports the hypothesis that bile salts, and CDCA in particular, can directly influence the phenotypic behaviour of pathogens within lung microbiomes.

**Figure 4:**
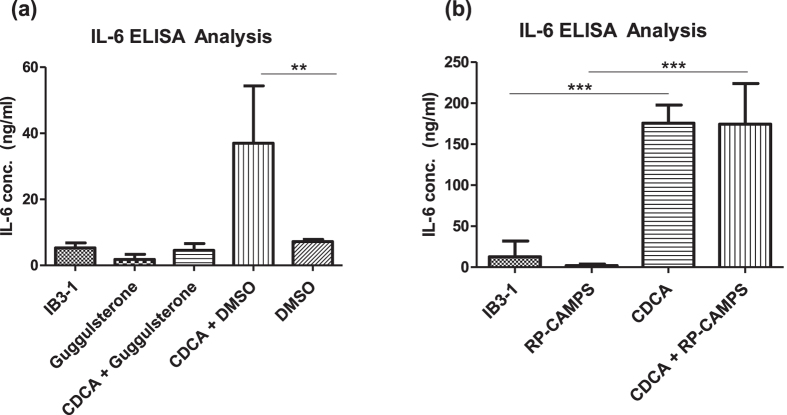
Bile salts trigger FXR-dependent pro-inflammatory cytokine production. (**a**) Induction of the pro-inflammatory cytokine IL-6 by CDCA involves the FXR receptor. (**b**) Induction of IL-6 occurs independent of the TGR5-PKA pathway. Data presented are the average of three independent biological replicates. Statistical analysis was performed by one-way ANOVA and Bootstratio (*p ≤ 0.05, **p ≤ 0.005, ***p ≤ 0.001).

The implication of CDCA in modulating chronic microbial behaviour was particularly relevant in light of the fact that we have recently shown that it increases pro-inflammatory cytokine production in CF-affected IB3-1 lung epithelial cells^14^. The induction of pro-inflammatory cytokines is known to pre-dispose the lung to chronic colonisation, which itself is promoted by exposure to CDCA. Furthermore, we have also demonstrated that both CDCA and PQS, which is induced in *P. aeruginosa* in response to bile, can destabilise the α subunit of Hypoxia-Inducible Factor (HIF)-1, a key host regulator involved in the hypoxic and immune response that is required for resolution of acute inflammation in intestinal cells^13^,^14^,^40^. Although several bile acid receptors exist in host cells, the redox signature of the microbial response to bile strongly implicated the nuclear Farnesoid X Receptor (FXR) for which CDCA has previously been shown to be a physiological ligand^41^,^42^. Addition of an FXR antagonist inhibitor, guggulsterone, led to abolition of IL-6 induction in the presence of CDCA (Fig. 4a). In contrast, IL-6 induction occurred independent of the membrane associated bile acid TGR-5 receptor, as seen with the addition of a Protein Kinase A inhibitor RP-CAMP (Fig. 4b). Conversely, bile-induced HIF-1α destabilisation occurred independently of FXR and TGR-5 (Supplementary Fig. S4). The dual action of CDCA on both pathogen and host may underpin the self-perpetuating cycles of increased colonisation and inflammation associated with *P. aeruginosa* infected aspirating patients.

### Bile aspiration correlates directly with colonisation by *P. aeruginosa* and other Proteobacterial pathogens in the CF lung

**Figure 5:**
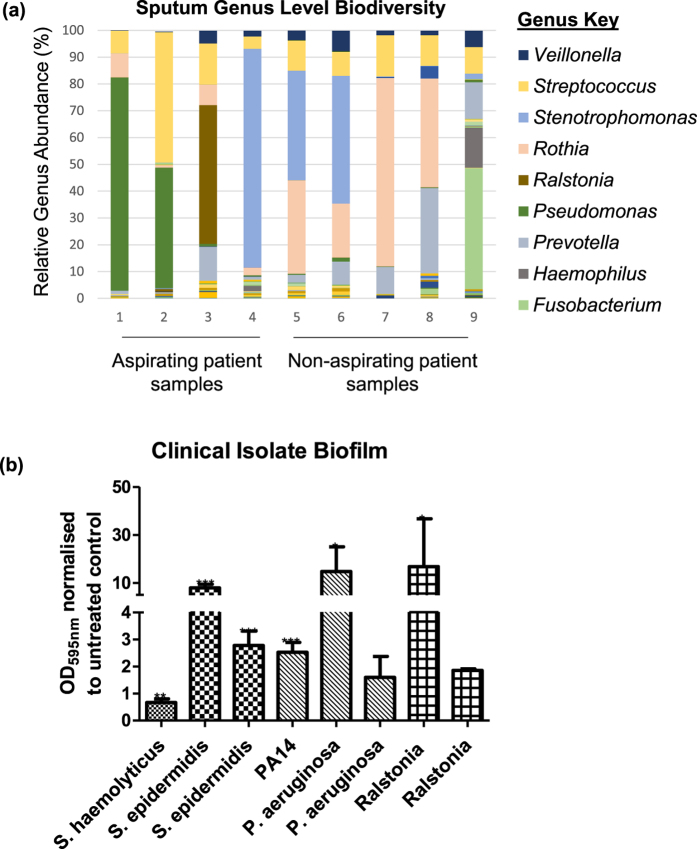
Bile acids in sputum correlate with Proteobacterial dominance. (**a**) Increased relative abundance of Proteobacterial pathogens was observed in aspirating samples (1–4) compared to non-aspirating (5–9). Samples 1 and 2 were taken 2 months apart from the same aspirating and suggest an exacerbation with Streptococcus. Samples 5 and 6, which were taken 3 months apart from a non-aspirating patient, reveal a stable and relatively rich microbiome of a non-aspirating patient. The x-axis numbers refer to the ID Nos. in Tables S3 and S4. (**b**) Biofilm analysis of clinical isolates from microbiome study performed on multi-well plates and quantified by crystal violet staining. Data presented is the average of at least three independent biological replicates. Statistical analysis was performed by Bootstratio (*p ≤ 0.05).

Ultimately, bacterial pathogens exist within the lung in polymicrobial communities, now commonly referred to as microbiomes. The individual response of members of the microbiome to external factors determines the flux and dynamics of these communities. Furthermore, rather than comprising of clonal populations, there has been a growing appreciation in recent years of the heterogeneity that exists, both at a genotypic and phenotypic level, in the lungs of respiratory disease patients^43^,^44^. Niche specific adaptation of clinical isolates has been reported, with mutations in key regulatory pathways discovered in several independent sequence-based studies^45^. Therefore, to investigate the longitudinal *in vivo* impact of aspirated bile in a clinical setting we performed bile acid profiling and longitudinal lung microbiome analysis (Supplementary Tables S3 and S4) on a cohort of paediatric patients (mean age 14.7 yr) from Cork University Hospital (CUH). The presence of bile acids correlated with reduced biodiversity (p = 0.0417) and richness (p = 0.0422) compared to sputum samples with low or undetectable levels of bile acids (Supplementary Fig. S5). Importantly, no statistical difference in age, antibiotic regime, or lung function (%FEV) was observed between the two bile stratified cohorts (Supplementary Table S3). Bile aspirating patient samples were colonised primarily by Proteobacterial pathogens which ranged from 50–90% of the total relative microbial abundance (Fig. 5a). In contrast, samples from non-aspirating patients exhibited increased diversity and only one patient carried more than 50% of any one organism, in this case *Rothia* (Fig. 5a). Longitudinal analysis was carried out on two patients and revealed an increase in *Streptococcus* over a four month period in the aspirating patient, while the non-aspirating patient retained a relatively diverse microbiome (Fig. 5a). The reduced biodiversity and colonisation by Proteobacterial pathogens has previously been described as a signature of the CF lung microbiome when compared with the lungs of ‘healthy’ non-CF patients^46^.

A collection of clinical isolates obtained from the paediatric CF microbiome cohort was screened for their phenotypic response to bile. Biofilm formation was assessed in clinical isolates of *Pseudomonas*, *Staphyloccoccus* and *Ralstonia*. Similar to *P. aeruginosa* PAO1 and PA14, biofilm formation was found to be markedly enhanced in the presence of bile in one of the *P. aeruginosa* clinical isolates tested, although to a greater degree suggesting a hyper-responsive phenotype. Biofilm was not significantly altered in another *P. aeruginosa* isolate (Fig. 5b). Two *S. epidermidis* isolates were also found to respond to bile by increasing biofilm formation, while a *S. haemolyticus* isolate exhibited a small but significant reduction in biofilm formation in response to bile. The phenotypic diversity at the species and genus level within the clinical isolate collection in response to bile suggests that either strain or isolate specific adaptations may occur in the CF lung. An extensive correlative analysis of bile acid profiles and clinical isolate behaviour in response to bile from the same biological samples may provide some clues as to the environmental driver of these adaptations.

## Discussion

The clinical management of respiratory disease remains an ongoing challenge. There is an urgent need to innovate and develop new options for the targeted prevention of microbial infection while avoiding the inevitable emergence of resistance that is the hallmark of broad spectrum antibiotic therapies. This requires a greater understanding of the molecular interactions underpinning the pathophysiology of respiratory disease, such as those arising from bile aspiration which has emerged in recent years as a major co-morbidity of chronic disease^9^,^10^.

**Figure 6:**
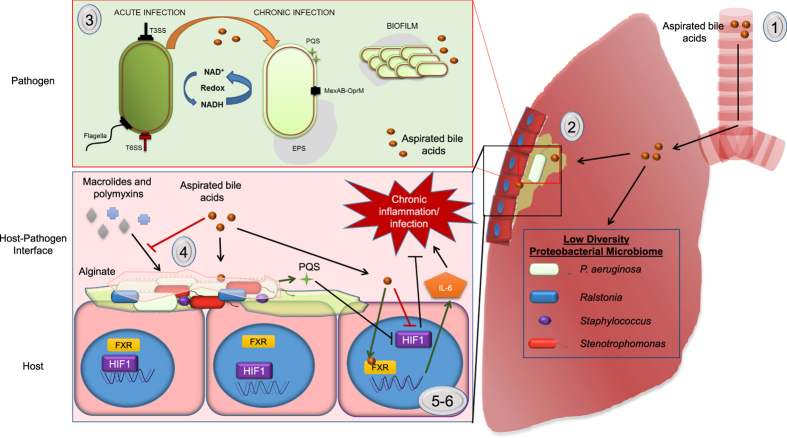
Overview of the bile response underlying chronic infection and chronic inflammation in respiratory disease patients. **(1)** The aspiration of bile acids into the lungs of patients with respiratory disease **(2)** correlates with a markedly reduced microbiome diversity in patients with CF. **(3)** Bile, with CDCA in particular, elicits a chronic lifestyle in several prominent respiratory pathogens. This includes a switch towards a biofilm lifestyle and suppression of virulence systems associated with the acute phase of infection. From a clinical perspective, **(4)** tolerance to the polymyxin and macrolide classes of antibiotics was observed in the presence of bile. In tandem with this, bile salts also **(5)** destabilised HIF-1 and **(6)** triggered FXR-dependent production of the IL-6 pro-inflammatory cytokine, potentially contributing to the chronic inflammation that characterises the pathophysiology of respiratory diseases such as CF.

The transcriptomic and phenotypic data emerging from this study suggests that redox flux linked to virulence control is a hallmark signature of microbial response to the bile acid challenge. Cycling of metabolic intermediates has previously been shown to influence virulence in *Pseudomonas aeruginosa*^47^. Furthermore, Takeuchi and co-workers found a strong correlation between the expression of the virulence associated small RNAs *rsmY* and *rsmZ* and the pools of 2-oxoglutarate, succinate, and fumarate in *P. fluorescens*^48^. Similar findings of redox controlled virulence have also been reported for other pathogens, including *Salmonella enterica*^49^, *Listeria monocytogenes*^50^, and *S. aureus*^33^. Indeed, the common thread of redox and oxidative stress perturbation in response to bile acid challenge links both the prokaryotic and eukaryotic responses (Fig. 6). It is possible that the synergies between both responses simply reflects the evolutionary linkages between both cell types, and the bile acid response represents a core cellular challenge inherent in lower and higher order organisms. Deciphering the linkage and the pathways involved will provide the foundation for future therapeutic interventions to target bile induced chronic infection and inflammation.

It now appears that specific bile acids create a chronic environment in the CF lung. Of the panel of the sodium salt of 12 bile acids identified to date in the CF lung, CDCA alone increased biofilm formation in the pathogen and induced FXR-dependent TGR5-independent IL-6 production in IB3-1 airway epithelial cells. The availability of FXR antagonists offers a possible clinical intervention in aspirating patients, although further *in vivo* work will be needed to underpin the therapeutic effectiveness of such an approach. Indeed, the implication of bile acids, and CDCA in particular, as a causative agent contributing to both chronic infection and chronic inflammation (Fig. 6) is consistent with the emergence of bile acids as mediators of a broad range of human diseases^51^. CDCA is a primary bile acid, which is synthesised *de novo* by the liver and can subsequently be conjugated with either taurine or glycine adjuncts, a process that is reversed by commensal bacteria. Conjugated CDCA did not promote biofilm formation in *P. aeruginosa* in this study, and was previously shown to be incapable of affecting HIF-1α stability^14^. Therefore, the role of the gut microbiome in establishing bile acid profiles within the lung may be a key factor contributing to the progression of lung disease. As the biological effects of individual bile acids begin to be considered independently of the total bile acid concentration, the importance of bile acid profiling of lung samples will become more critical to the clinical management of chronic respiratory disease.

The dual effects of bile acids on both host and pathogen is also likely to have indirect consequences for host-pathogen interactions in the lung. Previously, knockout of the IL-6 gene in a number of models has been shown to lead to decreased survival of the host cells following challenge with *Klebsiella pneumoniae* or *Mycobacterium tuberculosis*^52^,^53^. The protective role of IL-6 against bacterial infection is primarily through the recruitment and increased activation of neutrophil cells which leads to killing of bacteria^53^,^54^. There is evidence to suggest that bile aspiration results in increased production of both IL-6^14^ and neutrophils^12^ in affected patients, which together would likely challenge the infecting pathogen. In this context, the switch to a biofilm lifestyle would likely protect *P. aeruginosa* and other respiratory pathogens from this host challenge leading to persistence and the onset of chronic stage infection. HIF-1α destabilisation by bile acids, and CDCA in particular, is also interesting in this regard, given the finding that HIF-1 is required for the resolution of acute inflammation in intestinal cells^40^. On the basis of these collective findings, the impact of bile aspiration on the pathophysiology of respiratory disease warrants further clinical investigation.

In addition to promoting persistent infections and chronic inflammation, exposure to bile also results in tolerance to clinically relevant antibiotics. Until recently, the polymyxin class of antibiotics were described as a ‘salvage therapy’ for *P. aeruginosa* infected patients, with limited reports of resistance described. Indeed, inhaled tobramycin or colistin is recommended for early eradication to prevent establishment of chronic infection^55^. However, reports of transmissible resistance within *E. coli* and *K. pneumoniae* raise serious questions about the long term effectiveness of these drugs^39^. In this study, we have shown that bile increases *P. aeruginosa* tolerance to both colistin and polymyxin B, a phenotype that would further underpin the refractory nature and persistence of this pathogen in the lungs of patients with respiratory disease. Furthermore, tolerance to the clinically used macrolide erythromycin was also observed in response to bile. This is particularly relevant given the fact that macrolide antibiotics also have pro-kinetic activity, which would promote their use as anti-reflux and anti-aspiration therapies. Combined with the fact that *P. aeruginosa* is forming enhanced biofilms in the presence of bile, this increased antibiotic tolerance represents a serious clinical challenge where the pathogen is likely to be exposed to what are essentially sub-inhibitory concentrations of antibiotic. Rather than clearing the infection, these sub-lethal doses elicit a spectrum of virulence related responses, resulting in persistent and clinically refractive pathogens^56^,^57^. Therefore, targeting and preventing the aspiration of bile becomes a more pressing clinical need.

The within-species heterogeneity observed in the clinical isolate phenotyping is consistent with the growing number of reports of both phenotypic and genotypic variations among microbes in the CF lung^43^,^58^,^59^,^60^. We have recently shown that *S. aureus* clinical isolates also display a biofilm-inducing response to bile exposure, in contrast to the typed non-lung isolate NCDO949 (Ulliseweha *et al*., In Revision). Furthermore, a recent study by Sanchez and colleagues described selected bile acids that disrupt or reduce biofilm formation in a *P. aeruginosa wspR* mutant and in wild-type *Vibrio cholerae*^61^. Here, the different effects of bile acids on the *wspR* mutant likely reflect perturbation of cyclic-di-GMP levels, a second messenger whose production may be altered in response to bile based upon transcriptional induction of the *siaAD* system (Table S1). Perhaps it is unsurprising in light of the fact that the airway itself presents a heterogeneous environment to the pathogen, with gradients of oxygen limitation, inflammation, and microbial composition, all likely to impact on the niche-specific evolution of microbes in the host^62^. Recently, metabolic adaptation of *P. aeruginosa* in the host has been shown to directly impact on its interaction with *S. aureus*^63^. It follows, therefore, that the phenotypic heterogeneity that exists within the lung has consequences for the complex polymicrobial communities that inhabit human airways. Phenotypic heterogeneity among clinical pathogens also has consequences for the development of ‘pathogen specific’ therapies. Pathogen-specific neutralizing therapies have the benefit of avoiding the collateral damage caused by broad spectrum antibiotics to the polymicrobial communities that inhabit most niches. Current antimicrobial therapies tend to be non-pathogen-specific and there is evidence to suggest that the availability of relatively non-toxic broad-spectrum therapies has contributed to the emergence of resistance among both targeted and non-targeted microbes^64^. However, the heterogeneity that is emerging from independent studies suggests that these single-pathogen molecular based therapies will need to be appropriately designed to account for the adaptive diversity that persists in the target niche. It is notable that several of the mutations described are found in genes that are highly repressed in response to bile e.g. *hmgA* and *gltR*^45^,^65^, suggesting a possible adaptive advantage through mutation of these loci in an aspirating lung.

## Conclusion

This study has uncovered key insights into the molecular mechanism through which bile causes *P. aeruginosa* and other respiratory pathogens to switch from an acute virulent lifestyle to a chronic phase of infection. These chronic biofilm infections are largely refractive to antibiotic challenge, all the more significant in light of the increased tolerance to the polymyxin and macrolide classes of antibiotics observed in the presence of bile. These data strongly support the hypothesis that bile acids in the lungs are a major factor contributing to increased morbidity and eventual lung function decline in patients with respiratory disease. This adds to the growing evidence that clinical strategies preventing the transition of bile acids into the lungs could remove a key trigger for chronic infection and inflammation, thus improving patient health and quality of life. Understanding how aspirated bile causes chronic infection/inflammation will provide an unrivalled opportunity to develop innovative interventionalist therapies for the resolution of chronic respiratory disease. Currently, surgery in the form of laparoscopic Nissen fundoplication is the gold standard clinical option for treatment of reflux and associated aspiration^66^. However, the surgery is not without risk, and the incidence of aspiration in paediatric patients calls for less invasive options to be considered^67^,^68^. These may target bile aspiration at source, e.g. anti-GERD strategies such as the pro-kinetic macrolides which would reduce the extent of aspiration episodes and dual target pathogens in the lung^69^. Alternatively, bile acid sequestrants may prove effective in silencing the effects of aspirated bile on both pathogen and host, possibly delivered by inhalation to titrate out the bile acids in the lung. Finally, as molecular insights into the interaction between bile acids and the pathogen-host axis are further elucidated and understood, targeted personalised therapies become a more viable option. However, more extensive patient and systems based studies are required before the full extent of bile’s influence on the pathophysiology of respiratory disease is understood.

## Materials and Methods

### Bacterial culture

All cultures of *P. aeruginosa* were routinely grown in Tryptic Soy Broth (TSB) media at 37 °C with shaking at 180 rpm. Lab strains and clinical isolates were maintained on TSA or Casamino acid-Peptone-Glucose (CPG) agar at 37 °C. Mueller-Hinton (MH) agar and broth was used for antibiotic tolerance assays. Tetracycline (50 μg/ml) was added to media for maintenance of the *mexAB*-pMP220 reporter plasmid. The *mexAB* and *PA14_21210* TnM mutants used in the antibiotic tolerance assays were obtained from the non-redundant Harvard PA14 mutant library^70^. The concentrations of bile used in this current study have previously been shown to be sub-inhibitory to growth of *P. aeruginosa* and other respiratory pathogens^11^.

### RNA isolation and transcriptional analysis

Three independent cultures of *P. aeruginosa* strain PAO1 were exposed to 0.3% (w/v) bile (Sigma). Untreated and treated samples were grown shaking from OD_600 nm_ 0.025 to 0.8 at 37 °C. Subsequently 500 μl of culture was treated with 1 ml of RNAProtect Bacteria Reagent (Qiagen). Total RNA was extracted using the RNAeasy kit (Qiagen) according to the manufacturer’s instructions and DNase treated using TURBO DNase (Ambion). cDNA was synthesised using AMV reverse transcriptase (Promega), RNasin (100 U/μL) (Promega), Random Primers (0.5 μg/μL) (Promega) and 10 mM dNTPs (Promega). Real time primers were designed utilizing the Universal Probe Library Assay Design Center (UPL, Roche). RealTime PCR was conducted on a Chromo4 Continuous Fluorescence Detector (MJ Research) using FastStart TaqMAN Probe Master and probes from the Universal ProbeLibrary (UPL, Roche). All gene expression levels are presented relative to the housekeeping gene, *proC*.

Isolated RNA was sent to ATLAS Biolabs (Germany) for Affymetrix 3′ Expression Service analysis. RNA quantity and quality was assessed by Nanodrop and an Agilent Bioanalyser 2100. Production of the biotin-labelled cRNA, hybridisation, washing and scanning of the Affymetrix GeneChip (*Pseudomonas aeruginosa* Genome Array) was conducted at ATLAS Biolabs (Germany). GeneSpring GX software was used to analyse raw data to investigate expression changes in the treatment conditions. A Student’s paired t-test was carried out on the robust multiarray average (RMA)-normalised data generated from the microarray analysis using Gene Spring software to give a list of genes with altered expression greater than 1.5 and a p value ≤ 0.05.

### Transcriptome database design and analysis

Data representing all available transcriptomic datasets from *P. aeruginosa* were collected in excel format to generate a database of global profiling for this organism. A total of 250 datasets were collected and formatted to standardise the inputs for fold change of gene expression. All data was normalised to PAO1 nomenclature. A Pearson Correlation matrix was composed using XLSTAT (www.xlstat.com) software. Excel-based conditional formatting was applied to the data for visualisation. The R version 3.21 pheatmap software package was used to generate an association tree with the bile transcriptome dataset, based on the Pearson correlation matrix data (http://CRAN.R-project.org/package=pheatmap). All data utilised in this analysis and the appropriate references and metadata are provided in Table S2.

### Elastolysis assay

Elastolytic activity in *P. aeruginosa* was determined by an Elastin Congo Red (ECR) assay reported by Pearson and colleagues (1997)^71^, with modifications. Cells from mid-log phase cultures in PTSB (5% peptone 0.25% tryptic soy broth) were washed and resuspended in PTSB in the presence and absence of 0.3% bile to an O.D_600 nm_ of 0.05. After 21 hr at 37 °C with shaking, culture supernatants were filtered (0.45 μm pore-size filter). A 50 μl sample of the culture filtrate was added to tubes containing 20 mg of ECR and 1 ml of buffer (0.1 M Tris [pH 7.2], 1 mM CaCl_2_). Tubes were incubated for 18 hr at 37 °C with rotation and then placed on ice after 0.1 ml of 0.12 M EDTA was added. Insoluble ECR was removed by centrifugation at 13,000 rpm for 5 min. The supernatant was collected and the OD_495 nm_ was measured. Absorption due to pigments produced by *P. aeruginosa* was corrected for by subtracting the OD_495 nm_ of each sample incubated in the absence of ECR.

### Congo red binding assay

Analysis of polysaccharide production was performed using the protocol of Ghafoor and colleagues (2011)^72^. *P. aeruginosa* was inoculated at an OD_600 nm_ of 0.05 in 2 ml of PI medium (20 g peptone, 10 g K_2_SO_4_, 1.4 g MgCl_2_.6H_2_O, 25 mg of triclosan and 20 ml of glyercol per litre) in the presence and absence of 0.3% bile for 48 hr at 37 °C without shaking. The bacterial content, along with polysaccharides produced, was collected by centrifugation. The pellet was washed with PI medium and transferred into 2 ml microcentrifuge tubes. The pellet was resuspended in 1 ml of 20 mg/ml Congo red in PI medium and incubated for 90 min with shaking. Bacterial content and bound Congo red were removed by centrifugation at 13,000 rpm for 5 min. The supernatant was collected and the OD_490 nm_ was measured. The total Congo red percentage left in the supernatant was measured relative to the media control.

### Promoter fusion analysis

*P. aeruginosa* cultures carrying a *mexAB*-pMP220 promoter fusion plasmid was grown overnight in TSB broth supplemented with appropriate antibiotics. Cells were diluted to OD_600 nm_ of 0.05 in 20 ml and incubated at 37 °C with shaking at 150 rpm. A time course activity profiling was performed using cells recovered at the logarithmic and stationary phases of growth and β-galactosidase assays were performed as described previously^11^.

### Antibiotic tolerance assays

Overnight cultures of *P. aeruginosa* were prepared in MH broth and diluted to 0.5 MacFarland units. Uniform swabbing of cultures onto MH agar plates with or without 0.3% or 0.03% bile was followed by manual placing of antibiotic discs (Thermo Scientific) or E-strips (Thermo Scientific) on the surface of the inoculated agar. Plates were incubated overnight at 37 °C and scored by measurement of the diameter (disk assays) or visualisation (E-strip) of the bacterial growth. Growth kinetic experiments were performed on a BioScreen C plate reader (Oy Growth Curves Ab). Cells were inoculated from overnight cultures to OD_600 nm_ 0.05 and treated with increasing concentrations of polymyxin B or erythromycin, either in the presence or absence of bile. Growth kinetics were measured over 24 hr at 37 °C.

### Redox and Cellular Respiration Analysis

Reduction of tetrazolium violet (TV) to formazan in liquid cultures of *P. aeruginosa* was quantified as previously described^28^,^73^. For each strain, 200 μl of an overnight culture (adjusted to an OD_600 nm_ of 2.0) was added to 25 ml of TSB containing TV at a final concentration of 0.01 mg/ml. After incubation for 24 hr at 37 °C, cells were promptly assayed for total formazan production. Firstly, 5 ml of culture was harvested by centrifugation (5,000 × g, 5 min), and the supernatant was removed. Cells were then resuspended in 1.2 ml of dimethyl sulfoxide and centrifuged (5,000 × g, 5 min). Finally, the absorbance of the cell-free supernatant was measured at OD_510 nm_. Data interpretation was restricted to experiments in which the untreated sample value was ≥1.0.

### Biofilm assays

Bacterial cultures were incubated overnight and transferred into fresh TSB to an OD_600 nm_ of 0.05. Aliquots (1 mL) were transferred into 24-well plates and plates were incubated overnight at 37 °C in the presence and absence of bile or bile acids. Attachment/biofilm was evaluated by crystal violet staining after washing to quantify attached cells and measurements were taken spectrophotometrically at OD_595 nm_.

### Cell culture

IB3-1 cells (ATCC CRL-2777) are a bronchial epithelial cell line derived from a CF patient with CFTR *ΔF508/W1282X alleles*. IB3-1 cells were cultured in bovine serum albumin-collagen-fibronectin-coated flasks using LHC-8 medium (Invitrogen) supplemented with 10% (v/v) foetal bovine serum (FBS) and a combination of 100 units/ml penicillin and 100 μg/ml streptomycin (Invitrogen). Cell lines were purchased from the American Type Culture Collection (ATCC, LGC Standards). Cells were maintained at 37 °C in a humidified 5% CO_2_ atmosphere and used up to passage twenty. All experiments were performed on cells that had reached about 80% confluence after which the FBS was removed.

### ELISA analysis of HIF-1α and IL-6

Cytokine protein levels were measured in the supernatant (50 μl) of control IB3-1 cells and IB3-1 cells that were treated with DMOG, guggulsterone, RP-CAMPS, DMSO or CDCA (50 μM), or combinations thereof. A SingleAnalyte ELISArray kit was used for quantification of IL-6 (Qiagen), while HIF-1α levels were measured with the Human HIF-1α ELISA kit Simplestep (ab171577) according to the manufacturer’s instructions. A SpectraMax Plus384 (Molecular Devices, Sunnyvale, CA) was used to assay the absorbance and OD_450 nm_ data were corrected using OD_530 nm_ readings, as per manufacturer’s guidelines.

### LC-MS bile acid profiling

Profiling of the principal human bile acids was performed as described previously^10^. Twelve bile acid standards were used and resuspended in methanol to a concentration of 20 mM; CA, CDCA, DCA, LCA, UDCA, GDCA, TCDCA, TDCA, TCA, GCA, and TLCA were purchased from Sigma-Aldrich (Buchs, Switzerland). TUDCA was purchased from Calbiochem (Darmstadt, Germany). The twelve stock solutions were then pooled together to a concentration of 100 μM in methanol. All chemicals used were LC-MS grade. Duplicate samples were included in the analysis to ensure reproducibility and all analyses were performed blinded to patient data.

### Microbiome analysis

#### Patient cohort, ethics statement and sample processing

Sputum samples were collected from paediatric patients attending the CF clinic at Cork University Hospital, Ireland over a six month period. Ethical approval was granted by the Clinical Research Ethics Committee of the Cork Teaching Hospitals (CREC) for sputum collection and informed consent was obtained from all subjects with all patients/guardians signing consent forms for acquisition and analysis outlined in this study. All samples were handled in accordance with the approved guidelines. Sputum specimens were obtained by asking the patient to cough the specimen into a sterile universal container. Specimens were labelled with a unique study number. All sample collection was performed with the research group blinded to patient data. Sample processing was performed as described recently^10^.

#### 16S rRNA Microbiome Analysis

DNA extracted from sputolysin (Calbiochem) treated samples using a Puregene DNA Extraction Kit (QIAGEN) was quantified and standardised at a final concentration of 25 ng/μl. Samples were subsequently sent for 16S rRNA amplification and high throughput sequencing using the Roche FLX Genome Sequencer in combination with Titanium Chemistry at DNAVision (Belgium). Amplification of the V1-V3 region was achieved using the universal primers 518R 5′-ATTACCGCGGCTGCTGG-3′ and 27F 5′-AGAGTTTGATCCTGGCTCAG-3′. Amplicons were gel purified and concentrations for all samples were determined by the picogreen assay. A nested PCR approach was taken in light of the low target DNA abundance of the samples, consistent with previous reports^10^. Between 10089 and 19763 raw sequence reads were obtained from the nested amplification for each sputum sample (minimum read length of 430 bp) from which 4254–10028 passed QC analysis (Table S3). Each sequence passing QC was assigned to a family by the Ribosomal Database Project (RDP) classifier (v 2.1) with CE >80%. A minimum of 92.9% were assigned at the phyla level, while more than 91% of filtered 454 sequences were successfully classified down to the genus level. Richness and biodiversity indices based on Operational Taxonomic Units (OTUs) were extracted using mothur. The Chao1 index was used for richness estimation, related to the number of observed operational taxonomic units (OTUs). Biodiversity related to how uniformly the sequences are spread into the different observed OTUs, was estimated with the nonparametric Shannon formula. Both indices were evaluated at different distance unit cut-offs, to test different selectivity in the definition of OTUs.

### Statistical Analysis

Data for were analysed using Prism version 5.0 (GraphPad, San Diego, CA, USA) for statistical significance using one-way ANOVA or Student’s t-test. For normalised data presented as fold change, statistical analysis was performed using a Bootstratio algorithm (http://rht.iconcologia.net/stats/br/unique.html). For the microbiome analysis, a one-tailed unpaired t-test with Welch correction was applied. In all cases, p ≤ 0.05 was considered statistically significant.

## Additional Information

**How to cite this article**: Reen, F. J. *et al*. Bile signalling promotes chronic respiratory infections and antibiotic tolerance. *Sci. Rep.* **6**, 29768; doi: 10.1038/srep29768 (2016).

## Supplementary Material

Supplementary Information

Supplementary Table
