# Supplementary material for: Bile signalling promotes chronic respiratory infections and antibiotic tolerance
Source: Sci Rep. 2016 Jul 19;6:29768. doi: 10.1038/srep29768 (PMC4949476; doi:10.1038/srep29768)
Supplement: Supplementary Information [file srep29768-s1.pdf]

Bile signalling promotes chronic respiratory infections and antibiotic tolerance

F. Jerry Reen[1+], Stephanie Flynn[1+], David F. Woods[1], Niall Dunphy[1], Muireann Ní Chróinín[2], David Mullane[2], Stephen Stick[3], Claire Adams[1] and Fergal O'Gara[1,3,4*].

[1] BIOMERIT Research Centre, School of Microbiology, University College Cork, National University of Ireland, Cork, Ireland.

[2] Paediatric Cystic Fibrosis Unit, Cork University Hospital, Cork, Ireland.

[3] Telethon Kids Institute, Perth, Western Australia.

[4] School of Biomedical Sciences, Curtin Health Innovation Research Institute, Curtin University, Perth, WA 6102, Australia.

* f.ogara@ucc.ie.

[+]FJR and SF contributed equally to this work.

Table S1. Transcriptome profiling ≥ 1.5 fold changes in bile treated vs untreated.

Gene	Name	FC	Gene Description
PA0009	*glyQ*	1.5691702	glycyl-tRNA synthetase alpha chain
PA0013		1.9993705	conserved hypothetical protein
PA0024	*hemF*	1.5071424	coproporphyrinogen III oxidase, aerobic
PA0025	*aroE*	1.6457433	shikimate dehydrogenase
PA0061		1.7197822	hypothetical protein
PA0158	*triC*	1.527921	RND triclosan efflux transporter
PA0165		2.3387754	hypothetical protein
PA0169	*siaD*	2.6718676	diguanylate cyclase (GGDEF) domain
PA0170		2.435536	hypothetical protein
PA0171		2.2755358	hypothetical protein
PA0172	*siaA*	1.9316213	signal transduction protein
PA0195	*pntA*	5.698029	putative NAD(P) transhydrogenase, subunit alpha part 1
PA0196	*pntB*	6.0729065	pyridine nucleotide transhydrogenase, beta subunit
PA0200		1.6779655	hypothetical protein
PA0276		4.9222007	hypothetical protein
PA0293	*aguB*	1.6108009	N-carbamoylputrescine amidohydrolase
PA0305		1.7579664	acylhomoserine lactone acylase B
PA0316	*serA*	1.6069735	D-3-phosphoglycerate dehydrogenase
PA0352		1.7676547	probable transporter
PA0424	*mexR*	2.4193532	multidrug resistance operon repressor
PA0425	*mexA*	1.9427047	RND multidrug efflux membrane fusion precursor
PA0426	*mexB*	1.6745746	RND multidrug efflux transporter
PA0427	*oprM*	1.5616528	multidrug efflux outer membrane prote*in Opr*M precursor
PA0438	*codB*	1.7552981	cytosine permease
PA0482	*glcB*	2.3145316	malate synthase G
PA0506		12.926492	probable acyl-CoA dehydrogenase
PA0507		2.1321254	probable acyl-CoA dehydrogenase
PA0508		41.035236	probable acyl-CoA dehydrogenase
PA0520	*nirQ*	2.6049857	regulatory protein
PA0521		5.02969	probable cytochrome c oxidase subunit
PA0522		2.437193	hypothetical protein
PA0524	*norB*	11.786244	nitric-oxide reductase subunit B
PA0547		1.6460618	probable transcriptional regulator
PA0548	*tktA*	1.877121	transketolase
PA0559		1.875097	conserved hypothetical protein
PA0580	*gcp*	1.6231686	O-sialoglycoprotein endopeptidase
PA0607	*rpe*	1.627546	ribulose-phosphate 3-epimerase
PA0609	*trpE*	1.5326068	anthranilate synthetase component I
PA0642		1.5662348	hypothetical protein
PA0654	*speD*	2.553722	S-adenosylmethionine decarboxylase proenzyme
PA0665		1.741449	conserved hypothetical protein
PA0667		1.9470879	conserved hypothetical protein
PA0715		1.820787	hypothetical protein
PA0729		1.5332638	hypothetical protein
PA0750	*ung*	1.8501233	uracil-DNA glycosylase
PA0775		1.7557627	conserved hypothetical protein
PA0834		2.0055494	conserved hypothetical protein
PA0839		2.3640356	probable transcriptional regulator
PA0840		2.290152	probable oxidoreductase
PA0905	*rsmA*	2.0960267	global regulator protein
PA0916		1.5262712	conserved hypothetical protein
PA0917	*kup*	1.5049281	potassium uptake protein Kup

PA0945	*purM*	1.7995995	phosphoribosylaminoimidazole synthetase
PA0960		1.5693022	hypothetical protein
PA0975		2.0938315	probable radical activating enzyme
PA0976		2.3919008	conserved hypothetical protein
PA0996	*pqsA*	1.610995	AMP-dependent synthetase/ligase
PA1051		6.853995	probable transporter
PA1137		4.652861	probable oxidoreductase
PA1151	*imm2*	1.5625875	pyocin S2 immunity protein
PA1183	*dctA*	5.316506	C4-dicarboxylate transport protein
PA1198		2.0251155	conserved hypothetical protein
PA1228		1.8131458	hypothetical protein
PA1244		1.5826671	hypothetical protein
PA1296		1.5636294	probable 2-hydroxyacid dehydrogenase
PA1299		1.7093493	conserved hypothetical protein
PA1317	*cyoA*	2.316115	cytochrome o ubiquinol oxidase subunit II
PA1318	*cyoB*	2.6973588	cytochrome o ubiquinol oxidase subunit I
PA1319	*cyoC*	2.7420776	cytochrome o ubiquinol oxidase subunit III
PA1320	*cyoD*	3.0416453	cytochrome o ubiquinol oxidase subunit IV
PA1321	*cyoE*	2.38073	cytochrome o ubiquinol oxidase protein
PA1432	*lasI*	1.8146873	autoinducer synthesis protein
PA1554		1.518187	cytochrome c oxidase, cbb3-type, CcoN subunit
PA1596	*htpG*	5.018658	heat shock protein
PA1649		3.7435198	probable short-chain dehydrogenase
PA1687	*speE*	2.2749867	spermidine synthase
PA1736		1.934638	probable acyl-CoA thiolase
PA1748		5.4332643	probable enoyl-CoA hydratase/isomerase
PA1757	*thrH*	1.7832674	homoserine kinase
PA1772		1.8780539	probable methyltransferase
PA1787	*acnB*	1.7395196	aconitate hydratase 2
PA1828		2.5765126	probable short chain dehydrogenase
PA1829		2.7639565	hypothetical protein
PA1830		4.272302	hypothetical protein
PA1831		3.9432259	hypothetical protein
PA1834		1.5483704	hypothetical protein
PA1847	*nfuA*	2.1300695	Fe/S biogenesis protein
PA1959	*bacA*	1.6784254	bacitracin resistance protein
PA1970		2.1638315	hypothetical protein
PA1971	*braZ*	1.7112839	branched chain amino acid transporter protein
PA2063		1.778673	hypothetical protein
PA2120		2.5226676	hypothetical protein
PA2231	*pslA*	1.6181463	Psl exopolysaccharide biosynthesis
PA2232	*pslB*	1.7325034	Psl exopolysaccharide biosynthesis
PA2235	*pslE*	1.666137	Psl exopolysaccharide biosynthesis
PA2237	*pslG*	1.5830181	Psl exopolysaccharide biosynthesis
PA2251		1.5470891	hypothetical protein
PA2260		2.6197445	hypothetical protein
PA2272	*pbpC*	1.9574057	penicillin binding protein 3A
PA2282		1.712276	hypothetical protein
PA2285		3.5870645	hypothetical protein
PA2404		1.6703215	hypothetical protein
PA2453		1.524613	hypothetical protein
PA2524	*czcS*	1.5139198	signal transduction histidine kinase
PA2550		3.6004841	probable acyl-CoA dehydrogenase
PA2619	*infA*	1.5810775	initiation factor
PA2629	*purB*	2.0843713	adenylosuccinate lyase

PA2634	*aceA*	8.60934	isocitrate lyase
PA2653		2.838192	probable transporter
PA2662		6.3886952	conserved hypothetical protein
PA2663	*ppyR*	8.42526	*psl* and pyoverdine operon regulator
PA2705		3.5481186	hypothetical protein
PA2706		2.975919	hypothetical protein
PA2707		2.770848	hypothetical protein
PA2730		1.540463	hypothetical protein
PA2734		1.7171118	hypothetical protein
PA2735		1.8188722	probable restriction modification system protein
PA2740	*pheS*	1.5846324	phenylalanyl-tRNA synthetase alpha-subunit
PA2828		1.9770868	probable amino transferase
PA2849	*ohrR*	2.1531365	Mar-type transcriptional regulator
PA2854		1.6191936	conserved hypothetical protein
PA2876	*pyrF*	1.6983404	orotidine 5'-phosphate decarboxylase
PA2890	*atuE*	1.6387995	putative isohexenylglutaconyl-coA hydratase
PA2901		1.6329596	hypothetical protein
PA2951	*etfA*	2.38636	electron transport flavoprotein alpha subunit
PA2952	*etfB*	2.771388	electron transport flavoprotein beta subunit
PA2953		5.1129184	electron transport flavoprotein-ubiquinone oxidoreductase
PA2956		2.0893328	conserved hypothetical protein
PA2957		2.7422466	probable transcriptional regulator
PA2975	*rluC*	1.5661113	riobosomal large subunit pseudouridine synthase C
PA3001		1.5636102	probable glyceraldehyde-3-phosphate dehydrogenase
PA3006	*psrA*	3.473089	transcriptional regulator
PA3011	*topA*	1.5169479	DNA topoisomerase I
PA3012		3.0956378	hypothetical protein
PA3013	*foaB*	8.261979	fatty-acid oxidation complex beta subunit
PA3014	*faoA*	8.045702	fatty-acid oxidation complex alpha subunit
PA3039		1.6068201	probable transporter
PA3046		1.7461492	conserved hypothetical protein
PA3079		1.5431921	hypothetical protein
PA3080		2.2197092	hypothetical protein
PA3092	*fadH1*	10.941382	2,4-dienoyl-coA reductase
PA3136		4.564559	probable secretion protein
PA3142		1.5115281	integrase
PA3179		2.6249208	conserved hypothetical protein
PA3277		1.5552263	probable short chain dehydrogenase
PA3299	*fadD1*	2.3626924	long-chain-fatty-acid coA ligase
PA3310		1.5319793	conserved hypothetical protein
PA3312		1.6154325	probable 3-hydroxybutyrate dehydrogenase
PA3340		1.8735654	hypothetical protein
PA3397	*fprA*	1.5781447	oxidation-reduction process
PA3430		2.112962	probable aldolase
PA3436		3.7672973	hypothetical protein
PA3441		2.885145	probable molybdopterin-binding protein
PA3454		2.2919114	probable acyl-coA thiolase
PA3530	*bfd*	2.1058738	bacterioferretin-associated ferredoxin
PA3533	*grxD*	1.6208036	cell redox homeostasis
PA3564		1.5504247	conserved hypothetical protein
PA3604	*erdR*	1.8984143	response regulator
PA3608	*potB*	2.6690738	polyamine transport protein
PA3610	*potD*	3.1713614	polyamine transport protein
PA3636	*kdsA*	1.6291608	2-dehydro-3-deoxyphosphooctonate aldolase
PA3648	*opr86*	1.8314524	outer membrane protein

PA3673	*plsB*	1.5458521	glycerol-3-phosphate acyltransferase
PA3713	*spdH*	2.438359	spermidine dehydrogenase
PA3742	*rplS*	1.6403929	50S ribosomal protein L19
PA3810	*hscA*	1.9194839	heat shock protein
PA3815	*iscR*	1.97773	iron-sulfur cluster assembly transcription factor
PA3817		1.9172465	probable methyltransferase
PA3818	*suhB*	2.426292	extragenic suppressor protein
PA3832	*holC*	1.6676788	DNA polymerase III, chi subunit
PA3860		2.786317	probable AMP-binding enzyme
PA3876	*narK2*	2.4774816	nitrite extrusion protein 2
PA3915	*moaB*1	2.683761	molybdopterin biosynthetic protein B1
PA3925		6.362694	probable acyl-coA thiolase
PA3979		2.2068381	hypothetical protein
PA3980		1.5200603	conserved hypothetical protein
PA4053	*ribE*	1.5947592	6,7-dimethyl-8-ribityllumazine synthase
PA4055	*ribC*	2.0001235	rioboflavin synthase alpha chain
PA4139		2.0021958	hypothetical protein
PA4280	*birA*	1.6605364	cellular protein modification process
PA4291		1.5189809	hypothetical protein
PA4292		1.5532227	probable phosphate transporter
PA4385	*groEL*	2.1746135	60 kDa chaperonin
PA4386	*groES*	1.6020151	10 kDa chaperonin
PA4389		2.9126391	probable short chain dehydrogenase
PA4390		2.3305416	hypothetical protein
PA4404		1.5881593	hypothetical protein
PA4432	*rpsI*	2.0233817	30S ribosomal protein S9
PA4435		3.31337	probable acyl-CoA dehydrogenase
PA4519	*speC*	2.5638704	ornithine decarboxylase
PA4524	*nadC*	1.5230906	nicotinate-nucleotide pyrophosphorylase
PA4574		1.8216043	conserved hypothetical protein
PA4588	*gdhA*	4.843752	glutamate dehydrogenase
PA4602	*glyA3*	1.5978534	serine hydroxymethyltransferase
PA4615	*fprB*	1.7907255	ferredoxin reductase-type FAD binding protein
PA4621		1.950093	probable oxidoreductase
PA4625	*cdrA*	2.0494714	cyclic diguanylate-regulated TPS partner A
PA4637		1.6872619	hypothetical protein
PA4640	*mqoB*	1.8581218	malate:quinone oxidoreductase
PA4645		1.7798011	probable purine/pyrimidine phosphoribosyl transferase
PA4646	*upp*	1.5125525	uracil phosphoribosyltransferase
PA4676		1.7414601	probable carbonic anhydrase
PA4729	*panB*	1.6376442	3-methyl-2-oxobutanoate hydroxymethyltransferase
PA4730	*panC*	1.6072731	pantoate-beta-alanine ligase
PA4731	*panD*	2.025211	aspartate 1-decarboxylase precursor
PA4739		1.6100599	conserved hypothetical protein
PA4744	*infB*	1.610814	translation initiation factor IF-2
PA4747	*secG*	2.002634	secretion protein
PA4755	*greA*	1.554067	transcription elongation factor
PA4757		1.7081891	conserved hypothetical protein
PA4758	*carA*	2.2199163	carbamoyl-phosphate synthase small chain
PA4759	*dapB*	4.5988173	dihydrodipicolinate reductase
PA4768	*smpB*	1.660908	ssrA-binding protein
PA4770	*lldP*	6.846915	L-lactate permease
PA4771	*lldD*	6.3667874	L-lactate dehydrogenase
PA4817		1.8832077	hypothetical protein
PA4840		2.423288	conserved hypothetical protein

PA4855	*purD*	1.5000381	phosphoribosylamine -glycine ligase
PA4873		1.8353506	probable heat shock protein
PA4928		2.0750062	conserved hypothetical protein
PA5019		1.8728709	conserved hypothetical protein
PA5020		3.9215884	probable acyl-CoA dehydrogenase
PA5023		2.9872162	conserved hypothetical protein
PA5035	*gltD*	2.323267	glutamate synthase small chain
PA5048		1.5303047	probable nuclease
PA5072		1.859174	probable chemotaxis transducer
PA5076		1.7966675	probable binding protein component of ABC transporter
PA5118	*thiI*	1.7867571	thiazole biosynthesis protein
PA5119	*glnA*	2.0239024	glutamine synthetase
PA5125	*ntrC*	1.7712349	two component response regulator
PA5138		1.514509	hypothetical protein
PA5157		2.4622345	probable transcriptonal regulator
PA5158		2.5326378	probable outer membrane protein precursor
PA5159		3.614299	multdrug resistance protein
PA5192	*pckA*	2.1203341	phosphoenoylpyruvate carboxykinase
PA5215	*gcvT1*	1.6615047	glycine-cleavage system protein T1
PA5275		2.443186	conserved hypothetical protein
PA5296	*rep*	1.955691	ATP-dependent DNA helicase
PA5315	*rpmG*	1.5917709	50S ribosomal protein L33
PA5347		2.2524781	hypothetical protein
PA5407		2.249299	hypothetical protein
PA5430		1.5518764	hypothetical protein
PA5435		2.3119483	probable transcarboxylase subunit
PA5482		2.1968896	hypothetical protein
PA5503		1.51603	probable ATP-binding component of ABC transporter
PA5526		1.5122871	hypothetical protein
PA5530		2.2498746	C5-dicarboxylate transporter
PA5561	*atpI*	1.9720086	ATP-synthase protein I
Pae_tRNA_Ala		2.1793957	
Pae_tRNA_Gln		1.674767	
Pae_tRNA_Gly		1.5203607	
Pae_tRNA_His		1.8042011	
Pae_tRNA_Ile		1.868528	
Pae_tRNA_Leu		1.9550334	
Pae_tRNA_Lys		1.9264927	
Pae_tRNA_Thr		3.0495825	
Pae_tRNA_Val		1.7678549	
Downregulated genes			
PA0028		1.5948375	hypothetical protein
PA0039		2.143605	hypothetical protein
PA0048		2.94961	probable transcriptional regulator
PA0050		1.984994	hypothetical protein
PA0078	*tssL1*	2.1075292	Hcp secretion island I (HSI-I) type VI secretion system
PA0079	*tssK1*	1.6049535	Hcp secretion island I (HSI-I) type VI secretion system
PA0081	*fhA1*	1.7814412	Hcp secretion island I (HSI-I) type VI secretion system
PA0082	*tssA1*	2.4476979	Hcp secretion island I (HSI-I) type VI secretion system
PA0083	*tssB1*	2.0212882	Hcp secretion island I (HSI-I) type VI secretion system
PA0084	*tssC1*	2.1767128	Hcp secretion island I (HSI-I) type VI secretion system
PA0085	*hcp1*	1.9435418	Hcp secretion island I (HSI-I) type VI secretion system
PA0086	*tagJ1*	2.6898286	Hcp secretion island I (HSI-I) type VI secretion system
PA0087	*tssE1*	2.6551623	Hcp secretion island I (HSI-I) type VI secretion system

PA0088	*tssF1*	1.9184635	Hcp secretion island I (HSI-I) type VI secretion system
PA0089	*tssG1*	2.39119	Hcp secretion island I (HSI-I) type VI secretion system
PA0093	*tse6*	1.7975425	Hcp secretion island I (HSI-I) type VI secretion system
PA0094		1.6072897	hypothetical protein
PA0095		1.8542596	conserved hypothetical protein
PA0096		2.1944344	hypothetical protein
PA0099		5.3633327	hypothetical protein
PA0100		1.680287	hypothetical protein
PA0112		1.6479193	hypothetical protein
PA0122	*rahU*	3.615853	Hemolysin, aegerolysin type
PA0129	*bauD*	7.6909256	amino acid permease
PA0130	*bauC*	4.95721	oxopropanonate dehydrogenase
PA0131	*bauB*	5.0994606	beta-alanine biosynthetic protein
PA0132	*bauA*	9.548833	beta-alanine:pyruvate transaminase
PA0176	*aer2*	2.1970596	aerotaxis transducer
PA0178		1.7684873	probable two component sensor
PA0179		2.57306	probable two component response regulator
PA0250		1.566561	conserved hypothetical protein
PA0261		1.7094259	hypothetical protein
PA0262	*vgrG2*b	1.8797104	type VI secretion system Vgr family protein
PA0263	*hcpC*	4.5870194	secreted protein
PA0265	*gabD*	1.9571402	succinate-semialdehyde dehydrogenase
PA0266	*gabT*	2.0194545	4-aminobutyrate aminotransferase
PA0296	*spuI*	1.7034112	glutamylpolyamine synthetase
PA0297	*spuA*	2.749863	probable glutamine amidotransferase
PA0298	*spuB*	1.9352928	glutamylpolyamine synthetase
PA0299	*spuC*	1.9077505	Polyamine:pyruvate transaminase
PA0328	*aaaA*	1.7097926	arginine-specific autotransporter
PA0345		1.5220128	hypothetical protein
PA0433		1.5347764	hypothetical protein
PA0459		1.9906839	probable ClpA/B protease ATP binding subunit
PA0492		9.769155	conserved hypothetical protein
PA0493		12.164877	probable biotin-requiring enzyme
PA0494		12.537723	probable acyl-CoA carboxylase subunit
PA0495		14.1487875	hypothetical protein
PA0504	*bioD*	2.1558855	dethiobiotin synthase
PA0527	*dnr*	1.785652	transcriptional regulator
PA0563		2.0404708	conserved hypothetical protein
PA0602		2.9482942	probable binding component of ABC transporter
PA0730		20.264019	probable transferase
PA0744		2.6036005	probable enoyl-coA hydratase/isomerase
PA0745		2.601556	probable enoyl-coA hydratase/isomerase
PA0782	*putA*	2.399258	proline dehydrogenase
PA0783	*putP*	2.7099984	sodium/proline symporter
PA0789		2.135822	probable amino acid permease
PA0796	*prpB*	1.6505334	carboxyphosphonoenolpyruvate phosphonomutase
PA0798	*pmtA*	1.7103677	phospholipid methyltransferase
PA0818		1.538232	hypothetical protein
PA0852	*cbpD*	5.2773623	chitin-binding protein
PA0853		1.5048496	probable oxidoreductase
PA0855		2.013548	hypothetical protein
PA0865	*hpd*	9.271919	4-hydroxyphenylpyruvate dioxygenase
PA0870	*phhC*	3.1358166	aromatic amino acid aminotransferase
PA0871	*phhB*	2.0197833	pterin-4-alpha-carbinolamine dehydratase
PA0872	*phhA*	1.843872	phenylalaine-4-hydroxylase

PA0887	*acsA*	8.322072	acteyl-coenzyme A synthetase
PA1015		1.5982444	probable transcriptional regulator
PA1069		1.9617678	hypothetical protein
PA1073	*braD*	1.8554968	branched chain amino acid transport protein
PA1130	*rhlC*	2.571125	rhamnosyltransferase 2
PA1131		2.6840458	probable major facilitator superfamily transporter
PA1170		3.169312	conserved hypothetical protein
PA1173	*napB*	1.9207349	cytochrome c-type protein precursor
PA1175	*napD*	1.769999	protein of periplasmic nitrate reductase
PA1176	*napF*	2.807975	ferredoxin protein
PA1177	*napE*	2.4080005	periplasmic nitrate reductase protein
PA1293		3.1160045	hypothetical protein
PA1336	*aauS*	1.5600288	signal transduction histidine kinase
PA1338	*ggt*	3.2098217	gamma-glutamyltranspeptidase precursor
PA1377		2.1422617	conserved hypothetical protein
PA1378		2.501052	hypothetical protein
PA1396		2.6544714	probable two component sensor
PA1415		1.5845275	hypothetical protein
PA1418		2.5522227	probable sodium:solute symport protein
PA1469		1.776468	hypothetical protein
PA1494		1.6269441	mucoidy inhibitor gene A
PA1511	*vgrG2*a	2.014915	type VI secretion system Vgr family protein
PA1538		1.5359794	probable flavin-containing monooxygenase
PA1565	*pauB2*	8.70774	FAD-dependent oxidoreductase
PA1591		2.0886438	hypothetical protein
PA1600		1.743144	probable cytochrome c
PA1609	*fabB*	2.2841575	beta-ketoacyl-ACP synthase I
PA1610	*fabA*	2.9927423	beta-hydroxydeacnoyl-ACP dehydrase
PA1611		1.7661884	hybrid sensor kinase
PA1612		1.7176058	hypothetical protein
PA1639		2.3704023	hypothetical protein
PA1647		1.8923525	probable sulfate transporter
PA1656	*hsiA2*	2.6254783	Hcp secretion island I (HSI-II) type VI secretion system
PA1657	*hsiB2*	3.1352513	Hcp secretion island I (HSI-II) type VI secretion system
PA1658	*hsiC2*	3.1408982	Hcp secretion island I (HSI-II) type VI secretion system
PA1659	*hsiF2*	3.9727623	Hcp secretion island I (HSI-II) type VI secretion system
PA1663	*sfa2*	4.714584	Hcp secretion island I (HSI-II) type VI secretion system
PA1664	*orfX*	4.7656765	Hcp secretion island I (HSI-II) type VI secretion system
PA1665	*fha2*	4.4706626	Hcp secretion island I (HSI-II) type VI secretion system
PA1666	*lip2*	2.7888627	Hcp secretion island I (HSI-II) type VI secretion system
PA1667	*hsij2*	3.4455078	Hcp secretion island I (HSI-II) type VI secretion system
PA1668	*dotU2*	3.3361132	Hcp secretion island I (HSI-II) type VI secretion system
PA1669	*icmF2*	3.4615045	Hcp secretion island I (HSI-II) type VI secretion system
PA1670	*stp1*	2.32084	Hcp secretion island I (HSI-II) type VI secretion system
PA1691	*pscT*	1.7019385	translocation protein in type three secretion
PA1692		1.9261502	probable translocation protein in type three secretion
PA1693	*pscR*	1.9027892	translocation protein in type three secretion
PA1694	*pscQ*	2.4248476	translocation protein in type three secretion
PA1698	*popN*	1.8647052	type three secretion outer membrane protein precursor
PA1699	*pcr1*	2.2103293	negative regulator of protein secretion
PA1700	*pcr2*	2.8731177	type three secretion protein
PA1701	*pcr3*	2.8128605	type three secretion protein
PA1703	*pcrD*	2.2703972	type three secretory apparatus protein
PA1705	*pcrG*	2.268675	type three secretion regulator
PA1706	*pcrV*	2.441468	type three secretion protein

PA1707	*pcrH*	2.5031915	regulatory protein
PA1708	*popB*	3.310198	translocator protein
PA1709	*popD*	3.3381078	translocator outer membrane protein precursor
PA1710	*exsC*	3.9618962	exoenzyme S synthesis protein C precursor
PA1711	*exsE*	3.2902606	type three secretion protein
PA1712	*exsB*	3.4639027	exoenzyme S synthesis protein B
PA1713	*exsA*	2.269879	transcriptional regulator
PA1714	*exsD*	2.0926607	negative regulator of protein secretion
PA1715	*pscB*	2.127073	type three export apparatus protein
PA1716	*pscC*	2.4568872	type three secretion outer membrane protein precursor
PA1718	*pscE*	1.9889266	type three secretion export protein
PA1719	*pscF*	2.4587705	type three secretion export protein
PA1720	*pscG*	2.1631007	type three secretion export protein
PA1721	*pscH*	2.7372322	type three secretion export protein
PA1722	*pscI*	2.2609296	type three secretion export protein
PA1725	*pscL*	2.0760052	type three secretion export protein
PA1732		1.9571875	conserved hypothetical protein
PA1759		1.9743236	probable transcriptional regulator
PA1760		3.1952362	probable transcriptional regulator
PA1761		2.0183575	hypothetical protein
PA1762		1.8740104	hypothetical protein
PA1774	*crfX*	2.6833508	hypothetical protein
PA1775	*cmpX*	1.6878084	conserved cytoplasmic membrane protein
PA1797		4.6390796	hypothetical protein
PA1818	*LdcA*	3.1492357	lysine-specific PLP-dependent carboxylase
PA1819		1.9307975	probable amino acid permease
PA1844	*tse1*	2.373152	Hcp secretion island I (HSI-I) type VI secretion system
PA1852		3.399476	hypothetical protein
PA1869		2.6705492	probable acyl-carrier protein
PA1894		2.0885706	hypothetical protein
PA1895		1.9763824	hypothetical protein
PA1897		2.7002308	hypothetical protein
PA1903	*phzE2*	3.8006172	phenazine biosynthesis protein
PA1904	*phzF2*	4.305888	probable phenazine biosynthesis protein
PA1905	*phzG2*	5.7028136	probable pyridoxamine 5'-phosphate oxidase
PA1950	*rbsK*	1.501952	ribokinase
PA1963		2.0812864	hypothetical protein
PA1984	*exaC*	59.43127	NAD+ dependent aldehyde dehydrogenase
PA1986	*pqqB*	2.1094584	pyrroloquinolone quinone biosynthesis protein B
PA1999	*dhcA*	31.309772	dehydrocarnitine coA transferase subunit A
PA2003	*bdhA*	2.8883147	3-hydroxybutyrate dehydrogenase
PA2008	*fahA*	34.142612	fumarylacetoacetase
PA2009	*hmgA*	54.90419	homogentisate 1,2-dioxygenase
PA2010		2.1838396	probable transcriptional regulator
PA2011	*liuE*	3.4714077	3-hydroxy-3-methylglutaryl-CoA lyase
PA2013	*liuC*	12.648429	putative 3-methylglutaconyl-CoA hydratase
PA2014	*liuB*	14.03458	methylcrotonyl-CoA carboxylase, beta subunit
PA2015	*liuA*	10.924191	putative isovaleryl-CoAdehydrogenase
PA2016	*liuR*	7.376461	regulator of *liu* genes
PA2019	*mexX*	1.5939317	RND multi drug efflux membrane fusio*n prote*in precursor
PA2041		2.957639	amino acid permease
PA2044		1.6550866	hypothetical protein
PA2066		3.08255	hypothetical protein
PA2069		5.337103	probable carbamoyl transferase
PA2080	*kynU*	1.9059906	kynureninase

PA2109		13.239002	hypothetical protein
PA2110		9.982603	hypothetical protein
PA2111		11.699324	hypothetical protein
PA2112		10.565109	conserved hypothetical protein
PA2113	*opdO*	15.40829	pyroglutamate porin
PA2114		12.587432	probable major facilitator superfamily transporter
PA2123		1.5930154	probable transcriptional regulator
PA2193	*hcnA*	2.0849488	hydrogen cyanide synthase
PA2194	*hcnB*	2.4813926	hydrogen cyanide synthase
PA2195	*hcnC*	2.7307336	hydrogen cyanide synthase
PA2196		1.603319	tetR family transcriptional regulator
PA2247	*bkdA1*	11.765008	2-oxoisovalerate dehydrogenase alpha subunit
PA2248	*bkdA2*	8.665485	2-oxoisovalerate dehydrogenase beta subunit
PA2249	*bkdB*	8.010507	branched chain alpha keto acid dehydrogenase lipoamide
PA2250	*lpdV*	10.541232	lipoamide dehydrogenase-Val
PA2304	*ambC*	1.5349882	AMB biosynthetic protein
PA2305	*ambB*	1.8090299	AMB biosynthetic protein
PA2358		7.733476	hypothetical protein
PA2378		1.5142914	probable aldehyde dehydrogenase
PA2423		1.9415448	hypothetical protein
PA2464		2.2603655	hypothetical protein
PA2503		1.558646	hypothetical protein
PA2537		1.8237628	probable acyltransferase
PA2538		1.863953	hypothetical protein
PA2539		2.16321	conserved hypothetical protein
PA2540		2.290972	conserved hypothetical protein
PA2541		2.353624	probable CDP-alcohol phosphatidyltransferase
PA2552		10.1244545	probable acyl-CoA dehydrogenase
PA2553		17.720968	probable acyl-CoA thiolase
PA2554		7.5938797	probable short chain dehydrogenase
PA2555		14.5416565	probable AMP-binding enzyme
PA2573		1.7526189	probable chemotaxis transducer
PA2624	*idh*	1.5125592	isocitrate dehydrogenase
PA2637	*nuoA*	1.7138935	NADH dehydrogenase I chain A
PA2682		1.5942562	conserved hypothetical protein
PA2684	*tse5*	2.1057823	protein secretion by the type VI secretion system
PA2685	*vgrG4*	1.5318247	Hcp secretion island I (HSI-I) type VI secretion system
PA2702	*tse2*	1.8557568	protein secretion by the type VI secretion system
PA2703	*tsi2*	2.2335365	Hcp secretion island I (HSI-I) type VI secretion system
PA2725		1.7536601	probable chaperone
PA2726		2.0102458	probable radical activating enzyme
PA2727		1.776595	hypothetical protein
PA2728		1.5782139	hypothetical protein
PA2752		1.720396	conserved hypothetical protein
PA2759		1.9633168	hypothetical protein
PA2761		2.1907022	hypothetical protein
PA2776	*pauB3*	2.2253318	FAD-dependent oxidoreductase
PA2780		2.649256	hypothetical protein
PA2781		1.8431194	hypothetical protein
PA2788		1.7293639	probable chemotaxis transducer
PA2790		1.6003789	hypothetical protein
PA2792		1.6244026	hypothetical protein
PA2867		1.7818938	probable chemotaxis transducer
PA2868		2.5292265	hypothetical protein
PA2883		1.7968694	hypothetical protein

PA2950	*pfm*	2.7392774	proton motive force protein
PA2967	*fabG*	1.761908	3-oxoacyl-(acyl-carrier-protein)reductase
PA2968	*fabD*	2.5239315	malonyl-CoA-(acyl-carrier protein)transacylase
PA3038		4.6687083	probable porin
PA3054		1.6859233	hypothetical protein
PA3068	*gdhB*	3.46976	NAD-dependent glutamate dehydrogenase
PA3089		1.915602	hypothetical protein
PA3111	*folC*	1.9460045	folylpolygultamate synthetase
PA3181		2.4894865	2-keto-3-deoxy-6-phosphogluconate aldolase
PA3182	*pgl*	2.4615443	6-phosphogluconolactonase
PA3183	*zwf*	1.8612951	glucose-6-phosphate 1-dehydrogenase
PA3186	*oprB*	5.547807	glucose/carbohydrate outer membrane protein precursor
PA3187		37.640736	probable ATP binding component of ABC transporter
PA3190		37.495556	probable binding protein component of ABC transporter
PA3191	*gtrS*	2.3700728	glucose transporter sensor
PA3192	*gltR*	2.8294287	two component response regulator
PA3193	*glk*	2.5788982	glucokinase
PA3194	*edd*	2.3691285	phospholgluconate dehydratase
PA3195	*gapA*	11.153055	glyceraldehyde 3-phosphate dehydrogenase
PA3222		1.8008807	hypothetical protein
PA3232		1.7556814	probable nuclease
PA3234		5.911297	probable sodium:solute symporter
PA3266	*capB*	2.062545	cold acclimation protein B
PA3267		2.013526	hypothetical protein
PA3271		2.1071553	probable two component sensor
PA3289		2.1252868	hypothetical protein
PA3294	*vgrG4*a	2.0742862	type VI protein secretion system complex
PA3326	*clpP2*	2.1203053	ATP-dependent Clp protease proteolytic subunit
PA3327		2.6560686	probable non ribosomal peptide synthetase
PA3330		2.478699	probable short chain dehydrogenase
PA3331		2.440465	cytochrome P450
PA3332		2.590828	conserved hypothetical protein
PA3333	*fabH2*	2.5470762	3-oxoacyl-(acyl-carrier-protein)synthase III
PA3335		3.2118356	hypothetical protein
PA3355		2.0893073	hypothetical protein
PA3356	*pauA5*	3.517199	glutamylpolyamine synthetase
PA3361	*lecB*	4.9500566	fucose-binding lectin PA-IIL
PA3362		1.601258	hypothetical protein
PA3363	*amiR*	2.094657	aliphatic amidase regulator
PA3365		2.497356	probable chaperone
PA3366	*amiE*	1.7536775	aliphatic amidase
PA3403		1.6708249	hypothetical protein
PA3471		2.838807	probable malic enzyme
PA3478	*rhlB*	1.5629418	rhamnosyltransferase chain B
PA3483		1.7651366	hypothetical protein
PA3484	*tse3*	2.1280935	Hcp secretion island I (HSI-I) type VI secretion system
PA3485	*tsi3*	2.2205892	Hcp secretion island I (HSI-I) type VI secretion system
PA3487	*tle5*	2.6968684	phospholipase D active protein
PA3488	*tli5*	2.0857	Hcp secretion island I (HSI-I) type VI secretion system
PA3511		1.7608668	probable short chain dehydrogenase
PA3516		8.26889	probable lyase
PA3519		3.9683826	hypothetical protein
PA3535		2.5359788	probable serine protease
PA3560	*fruA*	2.0949857	phosphotransferase system transporter fructose specific IIBC
PA3561	*fruK*	1.8346641	1-phosphofructokinase

PA3562	*fruI*	1.6994203	phosphotransferase system transporter enzyme I
PA3570	*mmsA*	1.8595018	methylmalonate-semialdehyde dehydrogenase
PA3581	*glpF*	3.1833086	glycerol uptake facilitator protein
PA3582	*glpK*	3.2480462	glycerol kinase
PA3583	*glpR*	1.5413748	glycerol-3-phosphate regulon repressor
PA3622	*rpoS*	1.8654066	sigma factor
PA3661		3.144587	hypothetical protein
PA3711		1.5237778	probable transcriptional regulator
PA3716		1.9169841	hypothetical protein
PA3723		1.7067646	probable FMN oxidoreductase
PA3779		1.6678269	hypothetical protein
PA3786		1.5774021	hypothetical protein
PA3791		2.0427399	hypothetical protein
PA3841	*exoS*	2.0348053	exoenzyme S
PA3842	*spcS*	3.8800082	specific Pseudomonas chaperone for ExoS
PA3843		2.883644	hypothetical protein
PA3850		1.5037203	hypothetical protein
PA3858		1.5532614	probable amino acid binding protein
PA3859		1.5182569	carboxylesterase
PA3906		1.6909509	hypothetical protein
PA3924		2.2293808	probable medium chain acyl-CoA ligase
PA3960		1.9433469	hypothetical protein
PA4024	*eutB*	9.765597	ethanolamine ammonia-lyase large subunit
PA4025		3.6506097	ethanolamine ammonia-lyase light chain
PA4040		1.6383013	hypothetical protein
PA4124	*hpcB*	1.984254	homoprotocatechuate 2,3-dioxygenase
PA4126		2.3422012	probable major facilitator superfamily transporter
PA4127	*hpcG*	1.919759	2-oxo-hept-3-ene- 1,7 dioate hydratase
PA4129		2.7885988	hypothetical protein
PA4130		2.168181	probable sulfite or nitrite reductase
PA4131		2.43465	probable iron-sulfur protein
PA4132		2.3007894	conserved hypothetical protein
PA4134		3.8384316	hypothetical protein
PA4198		2.1332781	probable AMP-binding enzyme
PA4211	*phzB1*	8.585621	probable phenazine biosynthesis protein
PA4217	*phzS*	5.672542	flavin-containing monooxygenase
PA4290		3.2010045	probable chemotaxis transducer
PA4296	*pprB*	1.8907439	two component response regulator
PA4312		1.676818	conserved hypothetical protein
PA4316	*sbcB*	1.8292457	exodeoxyribonuclease I
PA4318		1.994757	hypothetical protein
PA4320		1.7371818	hypothetical protein
PA4321		1.8312458	hypothetical protein
PA4348		1.9334596	conserved hypothetical protein
PA4397	*panE*	1.6094146	ketopantoate reductase
PA4441		1.5054107	hypothetical protein
PA4464	*ptsN*	1.5437149	nitrogen regulatory IIA protein
PA4489	*magD*	1.5218936	endopeptidase inhibitor protein
PA4490	*magC*	1.7564965	hypothetical protein
PA4495		1.8454317	hypothetical protein
PA4496		3.494979	probable binding protein component of ABC transporter
PA4497		3.9295037	probable binding protein component of ABC transporter
PA4498	*mdpA*	2.8390453	metallo-dipeptidase aeruginosa
PA4500		3.5027168	probable binding protein component of ABC transporter
PA4502		5.121125	probable binding protein component of ABC transporter

PA4503		3.6120512	probable permease of ABC transporter
PA4504		3.6977658	probable permease of ABC transporter
PA4505		2.5218732	probable ATP binding component of ABC transporter
PA4506		2.7894301	probable ATP binding component of ABC transporter
PA4521	*ampE*	1.6189638	antibiotic response protein
PA4550	*fimU*	1.7110652	type 4 fimbrial biogenesis protein
PA4551	*pilV*	1.9267063	type 4 fimbrial biogenesis protein
PA4583		1.598647	conserved hypothetical protein
PA4591		1.6394689	hypothetical protein
PA4592		1.6008954	probable outer membrane protein precursor
PA4603		1.5413858	hypothetical protein
PA4604		1.9048488	conserved hypothetical protein
PA4605		1.9862462	conserved hypothetical protein
PA4607		1.6575047	hypothetical protein
PA4660	*phr*	1.5931557	deoxyribodipyrimidine photolyase
PA4736		1.645675	hypothetical protein
PA4737		1.5593427	hypothetical protein
PA4846	*aroQ1*	3.026733	3-dehydroquinate dehydratase
PA4847	*accB*	2.2632728	biotin carboxyl carrier protein
PA4848	*accC*	2.665176	biotin carboxylase
PA4890	*desT*	2.196814	negative regulator of fatty acid metabolic process
PA4917		1.6167381	hypothetical protein
PA4978		2.09403	hypothetical protein
PA5015	*aceE*	2.4748633	pyruvate dehydrogenase
PA5016	*aceF*	1.9336244	dihydrolipoamide acetyltransferase
PA5033		1.9714878	hypothetical protein
PA5058	*phaC2*	2.070579	poly(3-hydroxyalkanoic acid) synthase 2
PA5062		1.645185	conserved hypothetical protein
PA5089	*pldB*	1.8433772	phospholipase D active protein
PA5112	*estA*	2.2522578	esterase
PA5113		1.7548673	hypothetical protein
PA5114		1.5116537	hypothetical protein
PA5152		1.6229354	probable ATP binding component of ABC transporter
PA5154		3.0212357	probable permease of ABC transporter
PA5155		1.8666053	amino acid ABC transporter *mem*brane protein
PA5167	*dctP*	2.2897437	C4 dicarboxylate transport
PA5168	*dctQ*	2.9596171	C4 dicarboxylate transport
PA5169	*dctM*	3.794928	C4 dicarboxylate transport
PA5174		10.5041275	probable beta-ketoacyl synthase
PA5184		1.951973	hypothetical protein
PA5208		2.355181	conserved hypothetical protein
PA5212		3.2855444	hypothetical protein
PA5219		1.9625584	hypothetical protein
PA5220		3.035381	hypothetical protein
PA5230		1.5580871	probable permease of ABC transporter
PA5235	*glpT*	1.5808283	glycerol-3-phosphate transporter
PA5266	*vgrG6*	2.6062305	type VI protein secretion system complex
PA5271		1.510594	hypothetical protein
PA5290		1.6901497	conserved hypothetical protein
PA5302	*dadX*	2.7517176	catabolic alanine racemase
PA5303		3.682151	conserved hypothetical protein
PA5304	*dadA*	3.0453987	D-amino acid dehydrogenase,small subunit
PA5312	*pauC*	2.6244156	aldehyde dehydrogenase
PA5313	*gabT2*	1.7187879	transaminase
PA5329		1.5208641	conserved hypothetical protein

PA5348		3.6003451	probable DNA binding protein
PA5355	*glcD*	2.6623282	glycolate oxidase subunit
PA5367	*pstA*	1.555267	membrane protein component of ABC phosphate transporter
PA5374	*betI*	2.1576834	transcriptional regulator
PA5380	*gbdR*	9.957009	putative amidotransferase
PA5396		1.9843612	hypothetical protein
PA5410	*gbcA*	3.55013	putative ring hydroxylating dioxygenase
PA5421	*fdhA*	1.9251707	glutathione-independent formaldhyde dehydrogenase
PA5428		1.5123227	probable transcriptional regulator
PA5429	*aspA*	4.8744445	aspartate ammonia-lyase
PA5458		1.5078423	hypothetical protein
PA5461		1.7103561	hypothetical protein
PA5510		1.8075719	probable transporter
PA5545		2.2842433	conserved hypothetical protein

Table S3. Patient data for paediatric patient cohort involved in microbiome study.

ID No.	Age	Sex	IV antibiotics[a]	PO antibiotics[a]	FEV%[b]	Mutation
**7**	17	M	28	56	90%	ΔF508/ ΔF508
**5,6**	11	M	30	CM + 56 days CP	85%	ΔF508/ ΔF508
**1,2**	16	M	21	CM + 28 days CP	86%	ΔF508/ ΔF508
**8**	16	M	14	CM	92%	ΔF508/ ΔF508
**4**	16	F	70	*Ma* + PO CP	52%	ΔF508/ ΔF508
**9**	19	M	28	100	84%	ΔF508/ ΔF508
**3**	8	F	49	42	96%	ΔF508/ ΔF508

[a] Number of days in 2012 CP – Ciprofloxacin PO – Oral antibiotics

[b] Predicted baseline CM – Chronic macrolide

	**%FEV**		**IV antibiotics**		**Age**	
	Aspirating	Non-Aspirating	Aspirating	Non-Aspirating	Aspirating	Non-Aspirating
	86	85	21	30	16	11
	96	90	49	28	8	17
	52	92	70	14	16	16
		84		28		19
**Average**	78	87.75	46.66667	25	13.33333	15.75
**ttest**	0.215231		0.073776		0.228917	

Table S4. Read Statistics from Next Generation Sequencing Study.

ID No.	Seq No.	nReads	nBases	lMed	lMax	lMin	lAvg	sAvg
**1**	DNA14003-002-L1	10089	4033943	446	527	30	399.84	31.09
**2**	DNA14003-005-L1	19162	7067466	403	861	30	368.83	30.56
**3**	DNA14003-003-L1	10494	4159148	430	530	30	396.34	30.72
**4**	DNA14003-004-L1	10298	4090740	448	547	31	397.24	30.30
**5**	DNA14003-006-L1	19730	7661685	451	737	32	388.33	30.98
**6**	DNA14003-007-L1	17390	6811025	432	847	34	391.66	30.39
**7**	DNA14003-008-L1	17158	6963414	459	777	35	405.84	31.21
**8**	DNA14003-009-L1	11588	4531129	445	1183	30	391.02	30.54
**9**	DNA14003-010-L1	18321	6724406	415	1093	29	367.03	30.51

nReads: number of reads; nBases: number of bases; lMed: median read length; lMax: maximum read length; lMin: minimum read length; lAvg: average read length; sAvg: average quality score (Phred score).

Supplementary Figure Legends

Fig S1. Transcriptomic profiling of the *P. aeruginosa* response to bile. (a) Several components of the *hmgA-maiA* pathway were shown to be significantly repressed in the presence of bile. Increased expression of the global virulence regulator *psrA* was also observed in the presence of bile, while the *gtrS-gltR-oprB* pathway was repressed. All transcriptional changes were congruent with the transcriptome dataset. Data presented is the mean of three independent biological replicates. Statistical analysis was performed by Student's t-test ($p < 0.05$). (b) Elastolytic activity was increased in the presence of bile, as seen by an increase in free congo red (OD_{495nm}) in the supernatant of treated cells. Data presented is the average of six independent biological replicates. Statistical analysis was performed by Student's t-test ($p<0.001$). (c) An increase in polysaccharide production was observed in the presence of bile, resulting in a decrease in free congo red (OD_{495nm}) in the supernatant of treated cells. Data presented is normalised to the media control and is the average of six independent biological replicates. Statistical analysis was performed by Student's t-test ($p<0.05$).

Fig S2. (a) Increased erythromycin tolerance occurs independent of the MexAB and PA5157-60 efflux systems. Although genes encoding both the *mexAB-OprM* and *PA5157-5160* efflux systems were upregulated in response to bile, the increased tolerance in the presence of erythromycin was observed in disk assays with all mutants indicating and independent phenotype. Data presented is the average of at least three independent biological replicates. Statistical analysis comparing treated to untreated samples was performed by paired Student's t-test (* $p \leq 0.05$, ** $p \leq 0.005$., *** $p \leq 0.001$). (b) Growth kinetics of wild-type and *PA14_21210* mutant strain in the presence of bile and polymyxin. The kinetics are comparable between wild-

type and mutant indicating that the increased tolerance in the presence of bile is not dependent on *PA14_21210*. Data is the mean of three independent biological replicates.

Fig S3. LCFA biofilm analysis. Data in each graph is the average of at least three independent biological replicates. Statistical analysis of fold-change was performed by Bootstratio analysis (*** $p \leq 0.001$).

Fig S4. Destabilisation of HIF-1α by the CDCA occurred independent of both FXR and the TGR5-PKA pathway as evidenced by reduced HIF-1α levels in the presence of either guggulsterone or RP-CAMPS. Statistical analysis was performed using One-way ANOVA with Bonferroni corrective testing (*** $p < 0.001$).

Fig S5. Microbiome analysis of the CUH paediatric cohort dataset. Both Shannon Index (biodiversity) and Chao1 (richness) were significantly reduced in samples from aspirating patients relative to those from non-aspirating patients. Statistical analysis was performed by unpaired Student's t-test with Welch's correction applied.

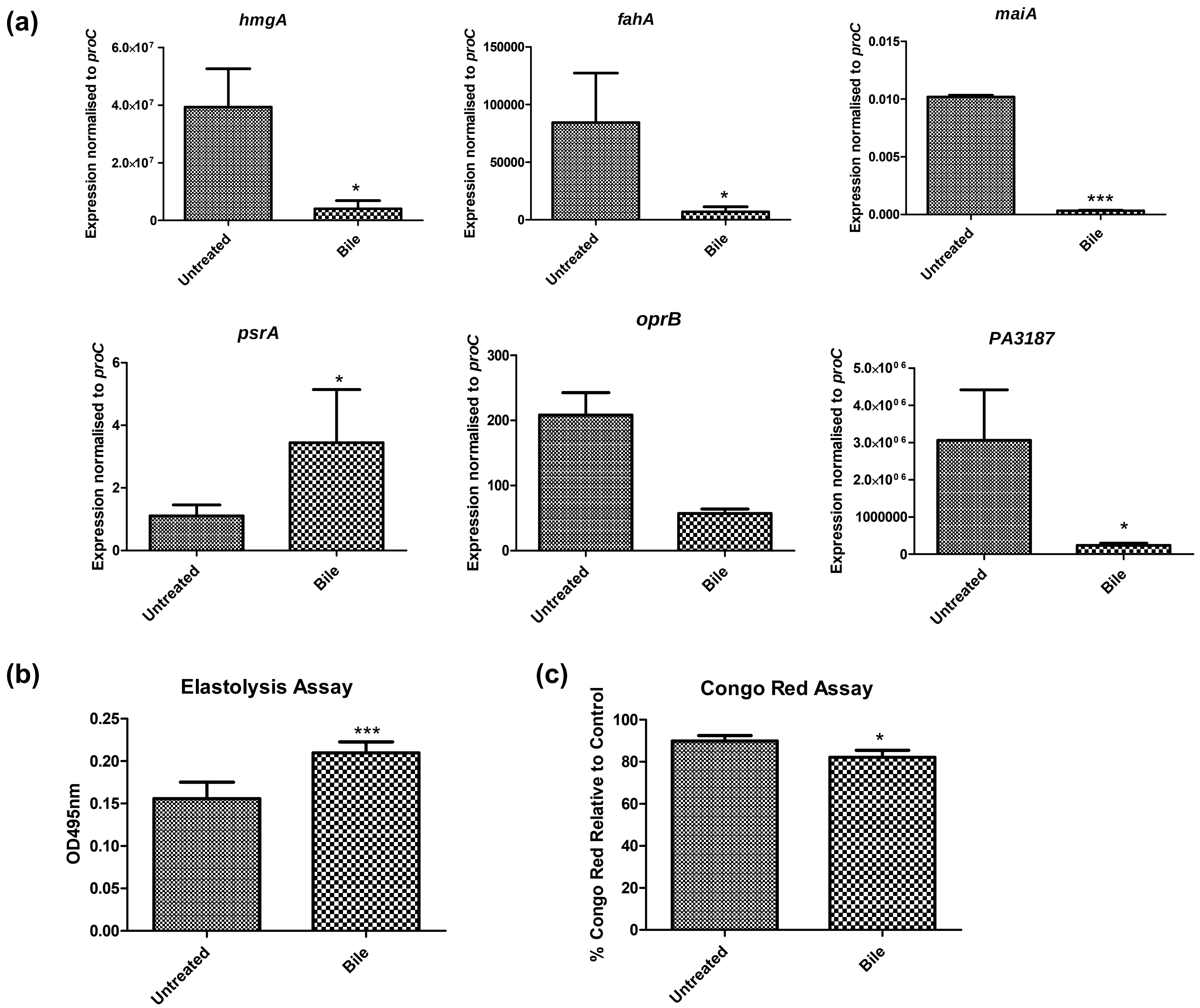
(a)
hmgA
Expression normalised to proC
6.0×10^7
4.0×10^7
2.0×10^7
0
Untreated
Bile
*
fahA
150000
100000
50000
maiA
0.015
0.010
0.005
0.000
***
psrA
6
4
2
oprB
300
200
100
PA3187
5.0×10^6
4.0×10^6
3.0×10^6
2.0×10^6
1000000
(b)
Elastolysis Assay
OD495nm
0.25
0.20
0.15
0.10
0.05
0.00
(c)
Congo Red Assay
% Congo Red Relative to Control
100
80
60
40
20

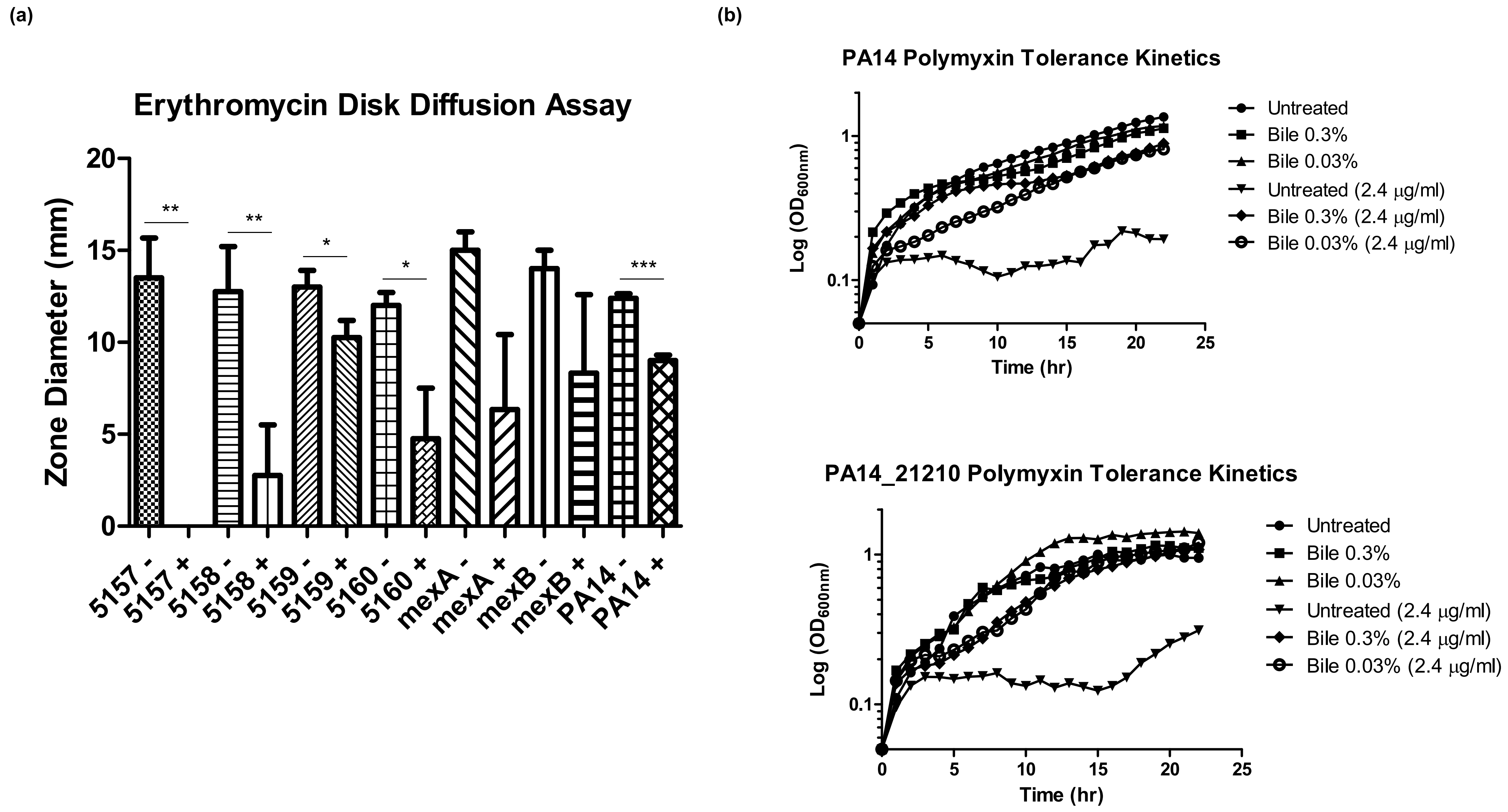
(a)
Erythromycin Disk Diffusion Assay
Zone Diameter (mm)
0
5
10
15
20
**
**
*
*
***
5157 -
5157 +
5158 -
5158 +
5159 -
5159 +
5160 -
5160 +
mexA -
mexA +
mexB -
mexB +
PA14 -
PA14 +
(b)
PA14 Polymyxin Tolerance Kinetics
Log (OD600nm)
0.1
1
Time (hr)
0
5
10
15
20
25
Untreated
Bile 0.3%
Bile 0.03%
Untreated (2.4 μg/ml)
Bile 0.3% (2.4 μg/ml)
Bile 0.03% (2.4 μg/ml)
PA14_21210 Polymyxin Tolerance Kinetics
Log (OD600nm)
0.1
1
Time (hr)
0
5
10
15
20
25
Untreated
Bile 0.3%
Bile 0.03%
Untreated (2.4 μg/ml)
Bile 0.3% (2.4 μg/ml)
Bile 0.03% (2.4 μg/ml)

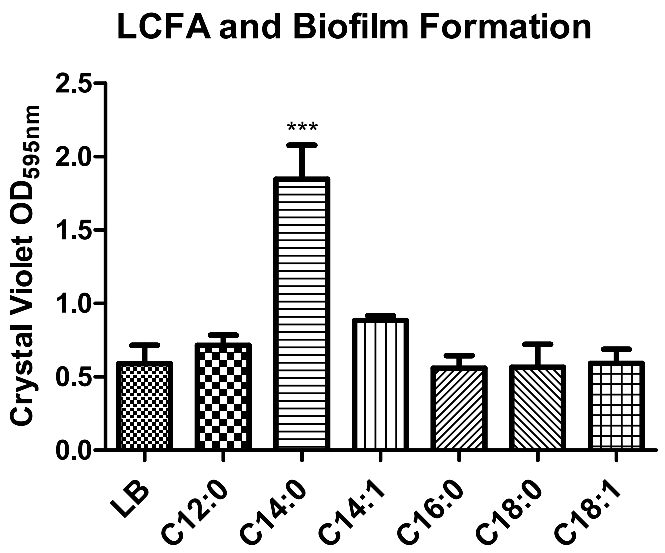
LCFA and Biofilm Formation
Crystal Violet OD595nm
2.5
2.0
1.5
1.0
0.5
0.0
***
LB
C12:0
C14:0
C14:1
C16:0
C18:0
C18:1

(a)

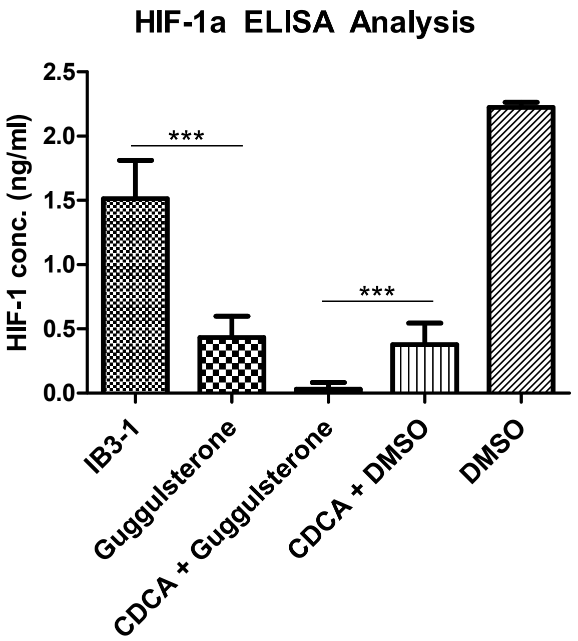
HIF-1a ELISA Analysis
HIF-1 conc. (ng/ml)
2.5
2.0
1.5
1.0
0.5
0.0
***
***
IB3-1
Guggulsterone
CDCA + Guggulsterone
CDCA + DMSO
DMSO

(b)

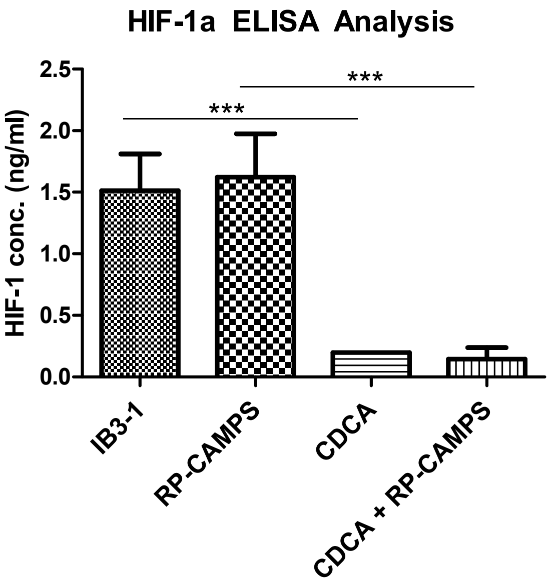
HIF-1a ELISA Analysis
HIF-1 conc. (ng/ml)
2.5
2.0
1.5
1.0
0.5
0.0
***
***
IB3-1
RP-CAMPS
CDCA
CDCA + RP-CAMPS

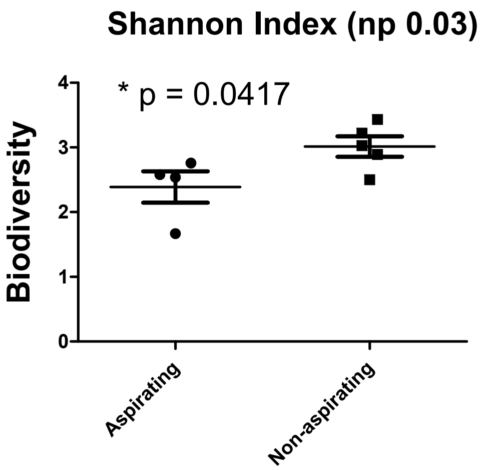
Shannon Index (np 0.03)
* p = 0.0417
Biodiversity
0
1
2
3
4
Aspirating
Non-aspirating

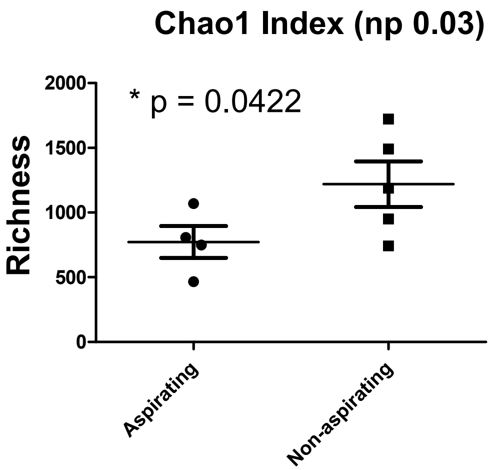
Chao1 Index (np 0.03)
* p = 0.0422
Richness
0
500
1000
1500
2000
Aspirating
Non-aspirating
